# Degradation of PGM and PGM-free Coatings on PEMWE Porous Transport Layers

**DOI:** 10.1021/acsami.4c22455

**Published:** 2025-03-11

**Authors:** Lukas Stein, Arne Dittrich, Dominic C. Walter, Patrick Trinke, Boris Bensmann, Richard Hanke-Rauschenbach

**Affiliations:** † Leibniz University Hannover, Institute of Electric Power Systems, Appelstraße 9A, Hannover 30167, Germany; ‡ Institute for Solar Energy Research Hamelin (ISFH), Am Ohrberg 1, Emmerthal 31860, Germany

**Keywords:** PEM, water electrolysis, coating, porous transport layer, degradation

## Abstract

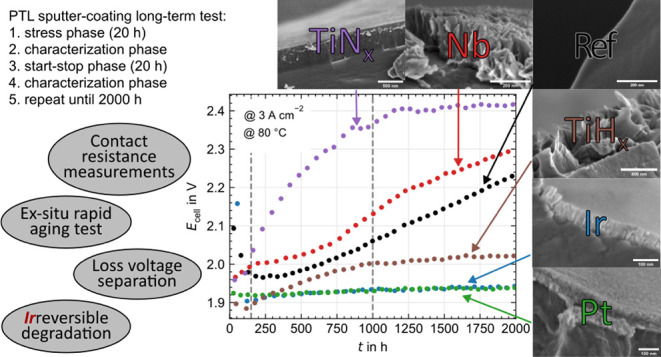

A good and long-term stable electrical contact between the porous anode transport layer (PTL) and the adjacent catalyst layer is essential for efficient polymer electrolyte membrane water electrolyzers. This study describes the extensive comparison of seven titanium passivation-protecting coatings using short- and long-term measurements for at least 2000 h. The measurements are supported by before and after scanning electron microscope investigations of cross sections, energy-dispersive X-ray spectroscopy, X-ray diffractometry of the coatings, contact resistance measurements, and ex situ rapid aging tests. Overall, iridium and platinum PTL coatings offer outstanding contact and excellent corrosion protection. Compared to the uncoated reference sample, platinum shows a 93% reduction in the overall degradation rate to 7 μV h^–1^ (at a current density of 3 A cm^–2^) over 5000 h and even reduces ohmic overvoltages over time in the first 2000 h. Interestingly, the interface to the flow field does not appear to be influenced by precious metal coatings and, hence, does not need to be coated. In contrast, niobium and titanium nitride PTL coatings under investigation do not provide an improvement compared to the uncoated reference but show dissolution and oxidation phenomena, respectively. Titanium hydride produced by hydrochloric acid improves the electrical contact and reduces degradation by 49% overall and 62% in terms of ohmic overvoltages compared to the uncoated reference. It also shows a saturation behavior in degradation with a stable rate of 23 μV h^–1^ in the second 1000 h of the measurement. Ex situ rapid aging tests additionally support the main trends. For all surface treatments, more detailed information about the occurring aging mechanisms and reversible overvoltages is obtained by separating the degradation rate into partial rates of the overvoltage mechanisms.

## Introduction

Polymer electrolyte membrane water electrolyzers producing green hydrogen have high requirements in terms of long-term stability and electrical conductivity of the components. The interaction of the porous transport layer (PTL) and catalyst layer (CL) at the anode interface is critical for longevity and minimization of ohmic overvoltages.^1^ Without a coating or other treatments, the PTL shows passivation and titanium oxide (TiO_*x*_) formation over time, strongly deteriorating the performance.^2^ Another essential point regarding the corrosion of PTLs are the different potential and pH value conditions on the PTL surface toward the flow field compared to the PTL-CL interface.^3^

Protective coatings are used to avoid passivation, as reviewed by Qiu et al.,^1^ Yuan et al.,^4^ Bautkinova et al.^5^ and Tao et al.^6^ Basically, a distinction can be made between precious metal and precious metal-free coatings. The former all show an improvement in electrical contact as a coating, including iridium (Ir),^7^ platinum (Pt),^8^ and gold (Au).^9,10^ Pt and Ir establish comparable, excellent corrosion resistance for over 4000 h reported by Liu et al.^11^ In contrast; a poor stability of Pt coating at high current densities has also been observed.^12^ Gold coatings have repeatedly shown dissolution during long-term measurements.^11,13^

Precious metal-free coatings include the treatment of PTLs with hydrochloric acid, which forms titanium hydride (TiH_*x*_), although this is technically not a coating. Nevertheless, TiH_*x*_ offers effective corrosion protection,^14^ and its long-term stability and linkability with Ir coatings have been investigated already.^15^ Niobium was used as a powder-based coating material in conjunction with stainless steel PTLs.^16,17^ Thermally produced titanium nitride (TiN_*x*_) shows slightly improved contact in the full cell,^9^ but long-term stability has not been proven yet. Recently, Ti_4_O_7_ and NiTiP,^18^ TiCN^19^ as well as TaPt^20^ and TaN,^21^ were also tested as coatings with minor effects on the electrical contact toward the catalyst layer.

This paper aims closing some specific and general gaps in the literature on PTL coatings. These combine to form a unique comprehensive comparison of seven PTL variants:

- Uncoated PTL as a reference measurement for all others
- Ir coated on both sides as a benchmark regarding conductivity and stability
- Ir coated on one side to evaluate the influence of the flow field side that might vary from the CL side due to different conditions
- Pt due to the ambiguous stability assessment compared to Ir
- Nb due to a lack of data on sputtered Nb layers on Ti-PTLs
- TiN_*x*_ especially for long-term stability evaluation
- TiH_*x*_ as another promising precious metal-free alternative

The study contains full cell reproducibility tests regarding the influence of electrical conductivity at the interface and at least 2000 h long-term measurements. A separation of the degradation rate into its ohmic, kinetic, and mass transport plus residual overvoltage parts is conducted, as well as the identification of reversible and irreversible shares. The coatings are characterized ex situ in terms of contact resistance, rapid aging, and layer composition before and after the long-term tests. The platinum group metals (PGM), Pt and Ir (also when coated on one side), show excellent contact and stability, while the PGM-free coatings Nb and TiN_*x*_ do not provide an improvement. The TiH_*x*_ treatment is the best PGM-free variant, significantly improving the cell performance and stability.

## Experimental Section

First, the preparation of the PTLs is described before the necessary information on the ex situ characterizations is given. Next, the setups for measuring the contact resistance and the rapid aging tests are presented. Finally, explanations of the electrochemical full cell setup and the data processing of the electrolysis measurements follow.

### Sample Preparation

The substrate for all full cell samples is a titanium fiber sintered PTL (Bekaert Bekipor) in 4 cm^2^ size with a thickness of 1 mm, which is cleaned in DI water in an ultrasonic bath for 15 min. The uncoated reference sample (denoted as Ref) is not subjected to further processing.

The deposition of the thin Ti–Ir (55 ± 4) nm, Ti–Pt (59 ± 4) nm, and Ti–Nb (210 ± 10) nm films (referred to as Pt, Ir, and Nb) as a double layer stack containing a (12 ± 4) nm thin Ti film underneath is created by magnetron sputtering. A set of samples coated only on one side with Ti–Ir will be referred to as Ir_1side_. The thin film deposition is conducted at room temperature using a cosputtering system from DREEBIT GmbH. The process background pressure is controlled to 5 × 10^–4^ mbar with an Ar background flow of 43 sccm. A power density of 5 W cm^–2^ is used at the planar magnetron sputter source (ION’X-4“HV).

The TiN_*x*_ (473 ± 11) nm film is prepared at room temperature by midfrequency magnetron sputter deposition double ring cathodes PPS-DR-220 in an LS900S sputtering system (Von Ardenne Anlagentechnik). The titanium target is made of a disk (*d* = 119 mm) and a circumferential ring (*d*_i_ = 125 mm, *d*_a_ = 220 mm) acting as alternating cathodes to prevent charging effects during reactive deposition. The power of the respective cathode is set to 1100 W for the ring and 200 W for the disc, corresponding to power densities of 4.3 W cm^–1^ and 2 W cm^–1^, respectively. The reactive deposition at a working pressure of 5 × 10^–3^ mbar is controlled by plasma emission monitoring, where the intensity of a spectral line of Ti during reactive sputtering (filter center line 453 nm) is monitored at 80% compared to the intensity of a nonreactive process. During the plasma emission monitoring assisted sputtering process, the reactive N_2_ gas flow is controlled between 1.9 and 2.5 sccm with a constant Ar background flow of 30 sccm. The film thickness is determined using cross-section scanning electron microscope (SEM) images of a single Ti fiber on top of the sample.

The preparation of the TiH_*x*_ PTLs is based on previous publications.^14,15^ First, hydrochloric acid (37%, Carl Roth) is heated to around 54 °C before etching the PTL for about 7 min. Directly afterward, the PTL is rinsed thoroughly with DI water. The surface of the hydrochloric acid etched samples (hereinafter referred to as TiH_*x*_) is not a coating, strictly speaking, but for the sake of clarity, it is treated as such.

### Ex Situ Characterization

Before electrochemical testing, cross-section images are taken with an S4800 SEM from Hitachi to analyze the thin film morphology of the samples. To achieve a smooth fracture edge for the cross-sectional images, a 0.25 mm thick Ti-PTL processed in parallel with the other samples is manually fractured in cryogenic liquid N_2_. This process exploits the metals brittleness at low temperatures of around −196 °C.

After the long-term tests, the degradation of the coatings is visualized in cross-section with a focused ion beam (FIB) SEM system (Zeiss Auriga). Pt is deposited locally for improved quality of the FIB cut through selected fibers. The FIB uses a Ga ion source at a current of 16, 4, and 2 nA. For the SEM images of the cross sections, the acceleration voltage is 10 kV using the in-lens secondary electron detector. For the TiH_*x*_ sample, images are additionally taken at an acceleration voltage of 3 kV using the in-lens energy selective backscattered electron detector, as dark areas in this view may indicate TiH_*x*_ due to the low density of hydrogen. In addition, energy-dispersive X-ray spectroscopy (EDX) mappings are recorded with an Oxford X-Max 80 detector to characterize the elemental distribution of the coating.

Grazing incidence X-ray diffraction (GIXRD) measurements with an incidence angle of ω = 5° are taken by the Empyrean X-ray diffractometer from PANalytical (Cu Kα, λ = 0.15406 nm). For the analysis of the GIXRD spectra, the software HighScore Plus version 3.0 is used in connection with the “Crystallography Open Database” (COD) as of March 2023.^22^

### Contact Resistance Measurement

The contact resistance measurements are carried out in a universal testing machine (Inspekt Duo M, Hegewald & Peschke) with gold-plated pressure plates. The resistance is measured by a potentiostat (Zahner Zennium Pro with power potentiostat PP242) with a 4-point measurement. The PTLs of the reproducibility tests (denoted as begin of test, BoT) and the long-term measurements (denoted as end of test, EoT) are rinsed with DI water, dried and placed between the pressure plates. The pressure is then gradually increased from 0.125 to 10 MPa, and at each measuring point, the voltage is measured at a current density of 1 A cm^–2^ and corrected by a blank measurement. The bulk resistance of the PTL is assumed negligibly small, and the contact resistance is calculated using Ohm’s law.

### Ex Situ Rapid Aging Test

The ex situ rapid aging test is carried out using a FlexCell corrosion measuring cell from Gaskatel. All samples are installed in the measuring cell as a working electrode and connected to the potentiostat (Biologic, EC-Lab V11.36). The potential of the sample is measured using a reference electrode (reversible hydrogen electrode (RHE) from Gaskatel). The corrosion measuring cell contains 0.5 mol L^–1^ sulfuric acid (H_2_SO_4_). The substrate material is a 3 × 5 cm^2^, 0.5 mm thick Ti-PTL (Bekaert Bekipor) with the different coatings (see section sample preparation) deposited on both sides. Because the corrosion measuring cell has to be sealed with an O-ring on the porous PTL, it is necessary that the PTL is additionally treated with wax in the area of the seal to avoid sulfuric acid leakage. Due to the lack of temperature stability of this wax, the measurements take place at room temperature. The measurement starts with a 4 h holding time at open circuit voltage (OCV) to establish an electrochemical equilibrium. After this, a linear sweep voltammetry (LSV) measurement is carried out, with the voltage at the working electrode increasing from OCV to 1.35 V vs RHE at a rate of 10 mV min^–1^. Here, the sample is kept for 12 h at an applied voltage of 1.35 V to ensure a high stress without significant gas evolution. The duration of the entire ex situ rapid aging test is approximately 19 h in total, depending on the value of the open circuit potential.

### Electrochemical Characterization

For electrochemical characterization, seven different 4 cm^2^ electrolysis cells (Fraunhofer ISE) with gold-plated flow fields and suitable frames (compression 5% anode, roughly 45% cathode) are used. The above-described Ti-PTLs are located on the anode side, in three repetitions for the reproducibility measurements and once for the long-term measurement, whereby samples from the reproducibility measurement of Pt and Ir_1side_ are reused for the long-term measurement. The catalyst coated membrane (CCM, Quintech) has a Nafion 115 membrane and CLs with 2 mg_Ir_ cm^–2^ and 1 mg_Pt_ cm^–2^. Before cell assembly, the CCM is placed in DI water for 60 min to ensure maximum hydration. The cathode gas diffusion layer (GDL) features a carbon paper (Freudenberg H23i2) with a thickness of 210 μm and hydrophobic treatment.

In-house built test benches are used for all measurements. Ion filters in the water circulation loop ensure high water quality (resistance >2 MΩ cm). The water temperature is controlled to 80 °C for all measurements with a flow rate of 80 and 25 mL min^–1^ for the reproducibility and long-term measurements, respectively. All measurements are carried out at ambient pressure. The clamping pressure of the cells is 6.25 MPa and is readjusted a second time only for long-term measurements after heating the cell and a 30 min break before starting the measurement. For capacity reasons, the measurements operate in several test benches, where the essential components are identical, and the results are therefore comparable. The long-term measurements of Ir, Ir_1side_, Nb, TiN_*x*_, and TiH_*x*_ are carried out on a multichannel test bench with a shared water circuit and heating, which is operated via a Shenchen Drifton LabN1-II pump with PharMed-BPT tubes. The test bench for the reproducibility measurements and the long-term measurements of Ref and Pt employs a Shenchen Drifton LabS3 pump with a PharMed-BPT tube. During the long-term measurements, a few short interruptions occurred that should be mentioned: Due to a power failure, the multichannel test bench interrupts for a few minutes after about 50 h (before the selected BoT time). After 2345 h for Pt and 1190 h for Ref, a 3 h interruption occurred due to a planned power shutdown while maintenance work on the building supply. Finally, temperature fluctuations can be observed in the Ir_1side_ measurement, especially from 1000 h on, which are due to a failure of the pump tube in this channel over time and cause this measurement to be terminated prematurely after 1250 h.

**Figure 1:**
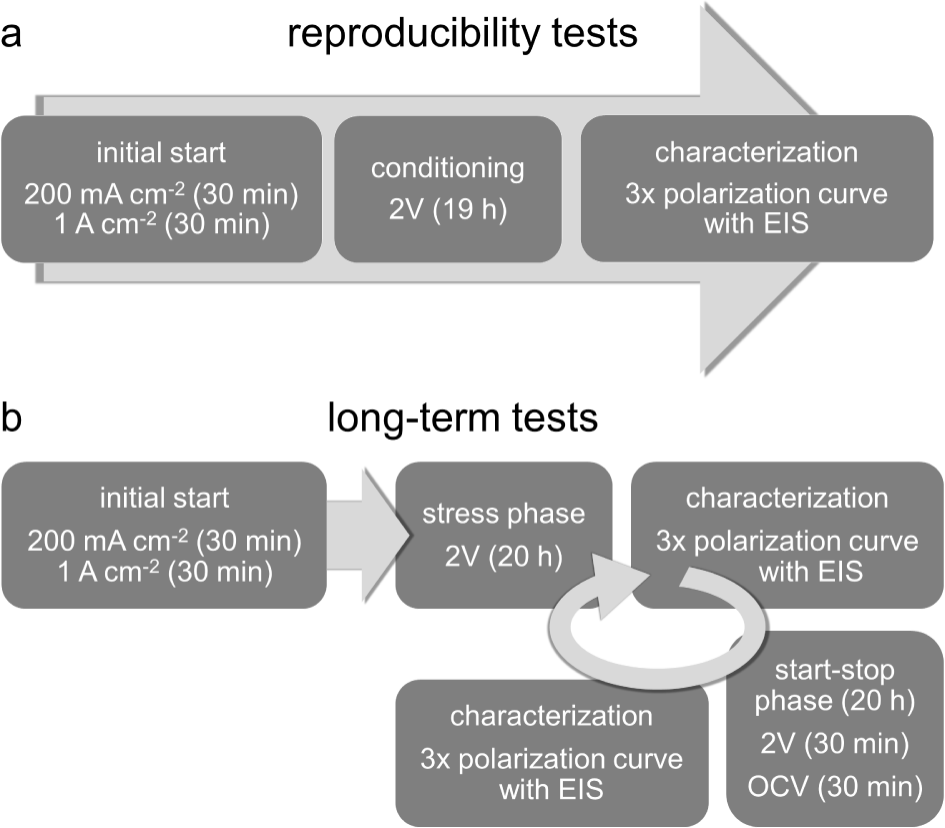
Potentiostat protocols for the reproducibility (a) and long-term (b) electrolysis measurements.

A BioLogic BCS-815 potentiostat controls power in all experiments; the test protocols are presented in Figure 1. All electrochemical impedance spectra (EIS) are measured from 10 kHz to 100 Hz with an AC of 10% of the DC amplitude. The reproducibility measurements are carried out three times each with new material in the same cell. According to Figure 1a, the protocol starts with a conditioning phase consisting of 30 min at a current density of 0.2 A cm^–2^, 30 min at 1 A cm^–2^, and 19 h at a voltage of 2 V with EIS measurements every 30 min. In the characterization phase, polarization curves from 10 mA cm^–2^ to 3 A cm^–2^ are recorded, with each current density step containing a 30 s hold step and an EIS measurement. Three polarization curves are recorded, of which only the third is used since most dynamic influences of the preceding operation point are neutralized.

The long-term measurements are each carried out once with a duration of 1250 h for Ir_1side_, 2000 h for Ref, Ir, Nb, TiN_*x*_, TiH_*x*_, and 5000 h for Pt. The Pt measurement then continues, without the data contributing to this study. As shown in Figure 1b, the measurement protocol consists of initial conditioning for 30 min at 0.2 and 1 A cm^–2^ each, after which four steps are repeated cyclical:

- A stress phase where 2 V are applied for 20 h with EIS every 30 min.
- A characterization phase analogous to the reproducibility measurements with three polarization curves from 10 mA cm^–2^ to 3 A cm^–2^, with each current density step containing a 30 s hold step and an EIS measurement.
- A start–stop phase with 20 h alternating between 2 V and OCV in 30 min intervals and EIS at the end of each interval.
- A second characterization phase, as described above.

The timestamps of the polarization curves are not identical for the different samples since the potentiostat can only record one EIS at a time, and the measurements are, therefore, shifted.

### Overvoltage Breakdown

For data evaluation, the cell voltage is plotted against the current density to visualize the polarization curve. In addition, the cell voltage *E*_cell_ is divided according to eq 1 into the pressure and temperature-dependent thermodynamic cell voltage  and ohmic (η_Ω_), kinetic (η_kin_), and mass transport plus residual overvoltages (η_mtx_).
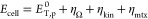
1

The ohmic overvoltage is calculated by the real axis intersection of the EIS’ Nyquist plot, the high frequency resistance (HFR) *R*_HFR_, multiplied by the current density *i* (eq 2). For some measurements, particularly long-term TiN_*x*_ measurements, there is no longer a real axis intersection of the EIS after a while. Thus, *R*_HFR_ contains an error since, in this case, the highest frequency (10 kHz) is assumed.
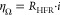
2

The HFR-free voltage *E*_iR-free_ is calculated by subtracting the ohmic overvoltage from the cell voltage (eq 3):
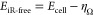
3

*E*_iR-free_ subtracted by  is then plotted against the logarithmic current density so that for small *i*, approximately only kinetic overvoltages occur (η_mtx_ is assumed to be very small in this region). The Tafel slope and exchange current density can be determined using a linear regression of the Tafel equation. Due to a slight drop in voltage at low current densities, the regression for the long-term tests is carried out in the range of 30–100 mA cm^–2^, while an interval of 10–100 mA cm^–2^ is chosen for the reproducibility measurements. The kinetic overvoltage η_kin_ is then calculated from the Tafel regression.

Finally, η_mtx_ can be determined using eq 1. In evaluating the long-term measurements, *E*_cell_ or individual overvoltages are plotted over time, and the degradation rate in μV h^–1^ is determined by linear regression.

## Results and Discussion

The part I of this section (“overall results”) contains a brief presentation of the coatings investigated and an overview of the most important results of the long-term measurements. The “deep dive” follows as part II, which includes the ex situ rapid aging measurements, reproducibility and contact resistance measurements, a breakdown of the overall degradation rate into partial degradation rates for individual overvoltages, an exploration of the reversible overvoltages, and the end of test (EoT) ex situ characterization featuring photos and SEM images including EDX as well as GIXRD.

### Part I: Overall Results

#### Cross-Sectional SEM Images

**Figure 2:**
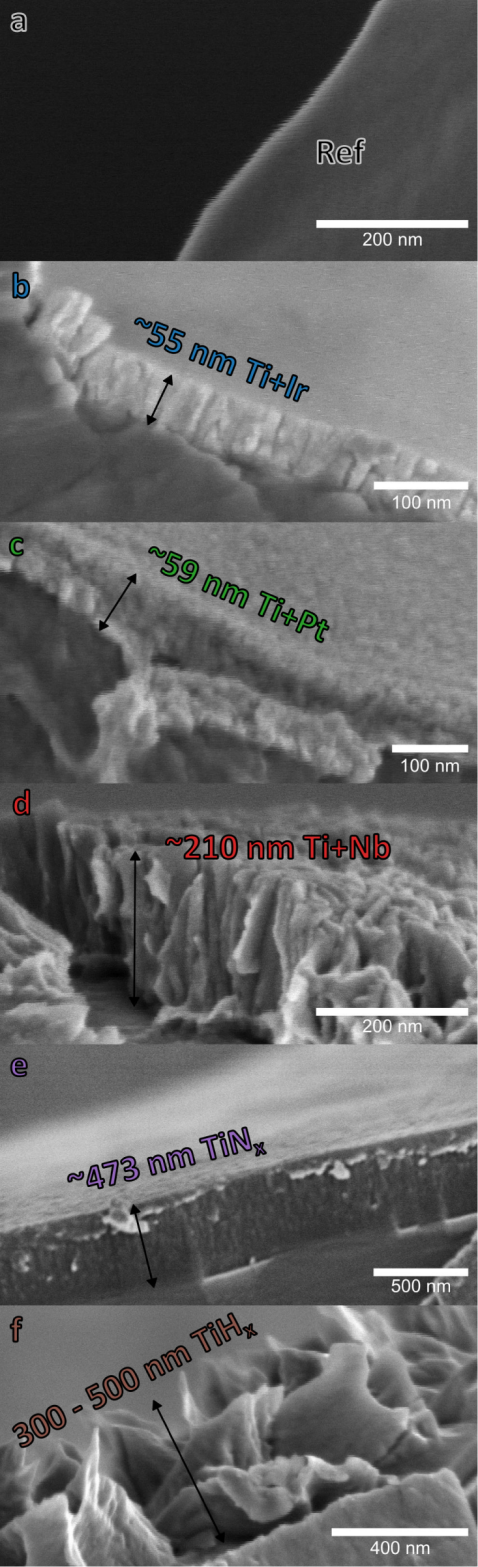
Liquid nitrogen fractured cross-section SEM images of PTLs Ref (a), Ir (b), Pt (c), Nb (d), TiN_*x*_ (e), and TiH_*x*_ (f).

Figure 2 shows the SEM images of the treated Ti-PTLs fractured in liquid nitrogen before testing intended to provide a basic insight into the general layer structure and thickness. Overview images and other magnifications are presented in Figure S1. This study includes a total of seven PTL configurations: uncoated reference (Ref, a), iridium double-side (Ir, b) and single-side coated (Ir_1side_, not shown here), platinum (Pt, c), niobium (Nb, d), titanium nitride (TiN_*x*_, e) and titanium hydride (TiH_*x*_, f).

The native TiO_*x*_ layer with a thickness in the low nm range is not recognizable for the Ref sample. The sputtered coatings (b–e) show typical columnar growth and an overall closed layer that only develops dislocations or cracks at the fracture edge. The PGM (Ir and Pt) grow fine-grained with a thickness of about 50–60 nm, including an ∼12 nm Ti interlayer to improve the adhesion. The Ti interlayer is also included underneath the Nb coating, which features a coarser structure and higher surface roughness (height approximately 210 nm). Co-sputtered TiN_*x*_ grows uniformly in columnar growth with a thickness of about 473 nm. In contrast to the sputtered coatings, the TiH_*x*_ layer, produced by hydrochloric acid etching, possesses spike structures with a height of several hundred nm. Underneath lies the Ti bulk material, brittle from the hydrogen (see Figure S1) but without recognizable effects on the measurement.

#### Long-Term Test Performance and Degradation Rates

The begin of test (BoT) time is 150 h due to run-in effects, and the end of test (EoT) is at 2000 h. For the Ir_1side_ sample, the EoT is at about 1250 h due to an unstable water flow of the test bench channel starting from about 1000 h, which causes irregularities in the cell temperature and, thus, the performance. The degradation rates are divided into two sections (<1000 h and >1000 h), as significantly different behavior occurs for some samples in the second half of the test. The description of the results follows in groups below: First, the PGM coatings, followed by the PGM-free ones.

**Figure 3:**
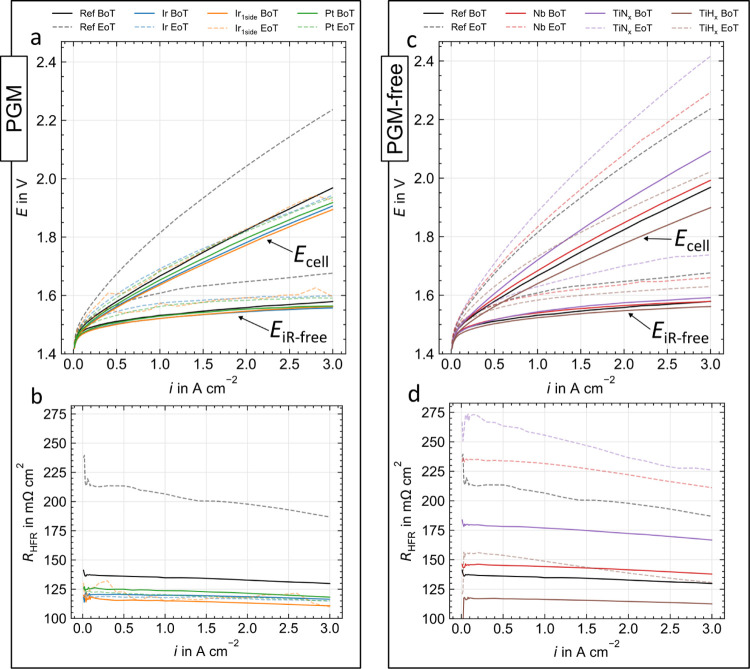
Begin (solid lines) and end of test (dashed lines) cell voltage with HFR-free voltage (a,b) and HFR (c,d) over current density for PGM (left side) and PGM-free (right side) PTL coatings, respectively. All measurements were carried out at 80 °C, ambient pressure, and with a Nafion 115 based CCM.

Figure 3 shows the polarization curves as well as HFR-free voltage (a) and the HFR (b) curves BoT (solid lines) and EoT (dashed lines) of the PGM coatings and the uncoated reference. For Ref, an increase in cell voltage is observed, as well as for HFR-free voltage and HFR. This finding suggests an apparent degradation in all essential overvoltages. The PGM coatings already reduce the cell voltage BoT compared to the uncoated reference sample.^7,11,23^ A reduction in HFR by about 15 mΩ cm^2^ indicates an improved contact resistance to the CL. An examination of the HFR-free voltage suggests comparable kinetics (at low current densities) and slightly reduced mass transport plus residual overvoltages (at high current densities). The coatings hardly age concerning the cell voltage, especially in comparison to Ref. Interestingly, HFR values for Pt and Ir have not increased but fallen slightly. The kinetics show noticeable degradation, indicating that the CL appears to be the bottleneck in this configuration.

**Figure 4:**
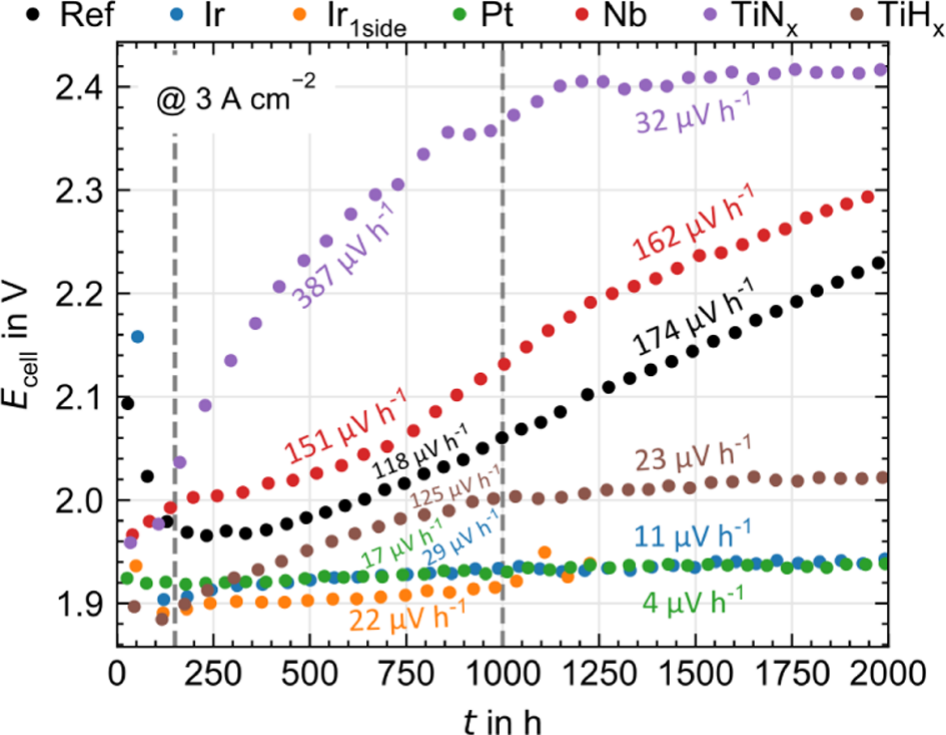
Cell voltage at 3 A cm^–2^ over time including degradation rates for PGM and PGM-free PTL coatings below and above 1000 h. All measurements were carried out at 80 °C, ambient pressure, and with a Nafion 115 based CCM.

Figure 4 shows the cell voltage values at 3 A cm^–2^ for the third polarization curves of the characterization after the stress phases over time, including the degradation rates in both halves of the test. The already described vital degradation of Ref can also be observed here, with an increasing degradation rate in the second half of the test (118 and 174 μV h^–1^, respectively), overall in a similar range to that in Rakousky et al.^2^ The PGM coatings have smaller degradation rates, which decrease in the second half of the test (29 to 11 μV h^–1^ for Ir and 17 to 4 μV h^–1^ for Pt). Pt degrades less than Ir (reduction in degradation compared to Ref by 93% and 86%, respectively), and thus proves its excellent stability analogous to Liu et al.^11^ and contrary to Rakousky et al.^12^ Interestingly, the interface toward the flow field does not seem to have a significant impact on performance: Ir_1side_ has a degradation rate of 22 μV h^–1^ in the first 1000 h, which is in the range of the double-side Ir coated PTL. The environment on this side of the PTL is not as corrosive as on the other side,^3,24^ which opens up the possibility of reducing the amount of PGM by 50%. It should be noted that a gold-plated flow field is used in the present study.

For the PGM-free coatings, the polarization curves and HFR-free voltages are presented in Figure 3c and the HFR curves in Figure 3d, again for BoT (solid lines) and EoT (dashed lines). The BoT performances differ: Ref and Nb show a comparable behavior, which varies from a powder-based Nb coating reported in the literature.^16^ TiN_*x*_ shows an increased voltage, while TiH_*x*_ significantly improves the performance. Both trends can be explained by an offset in the HFR upward (for TiN_*x*_) or downward (for TiH_*x*_), as previously reported in the case of TiH_*x*_.^14,15^ It is noteworthy that TiH_*x*_ shows even minimally better contacting than the PGM coatings. At EoT, the polarization curves remain in their order, and an evident layer degradation can be observed for Nb and TiN_*x*_, especially in the HFR. TiH_*x*_ degrades the least, resulting in a cell voltage reduction of about 200 mV at 3 A cm^–2^ compared to Ref at EoT. It is important to note that the HFR determination for the EoT TiN_*x*_ and Ref samples partially failed, because the real part of the impedance at 10 kHz is assumed to be the HFR (see Experimental Section).

In Figure 4, a uniform, high degradation rate can be observed for Nb over time (151 and 162 μV h^–1^) corresponding averaged to a 7% increased degradation rate compared to the uncoated reference, which in the EoT characterization will be attributed to a dissolution of the Nb layer. TiN_*x*_ and TiH_*x*_ establish a saturation effect in the degradation rate (387 to 32 μV h^–1^ and 125 to 23 μV h^–1^, respectively). The EoT characterization suggests an enrichment of oxygen in the coatings, which occurs especially in the first half of the test and significantly reduces further degradation. The effect is much more pronounced for TiN_*x*_, where the degradation rate is, on average, 43% higher than for Ref. The degradation rate of TiH_*x*_, on the other hand, is in the range of the PGM coatings in the second part of the measurement and reduces the degradation in total by about 49% compared to Ref.

In general, a correlation between the degradation behavior and HFR can be observed, which confirms the theory that the contacting of the CL is an essential component of the degradation and can be varied via the PTL coating. This connection is also visible in the HFR over time plot at a current density of 3 A cm^–2^ and the corresponding degradation rates (Figure S2). Furthermore, a decrease with increasing current density is observed for all HFR plots,^25,26^ which occurs more strongly in aged or poorly conductive layers.

### Part II: Deep Dive

#### Ex Situ Rapid Aging Test

**Figure 5:**
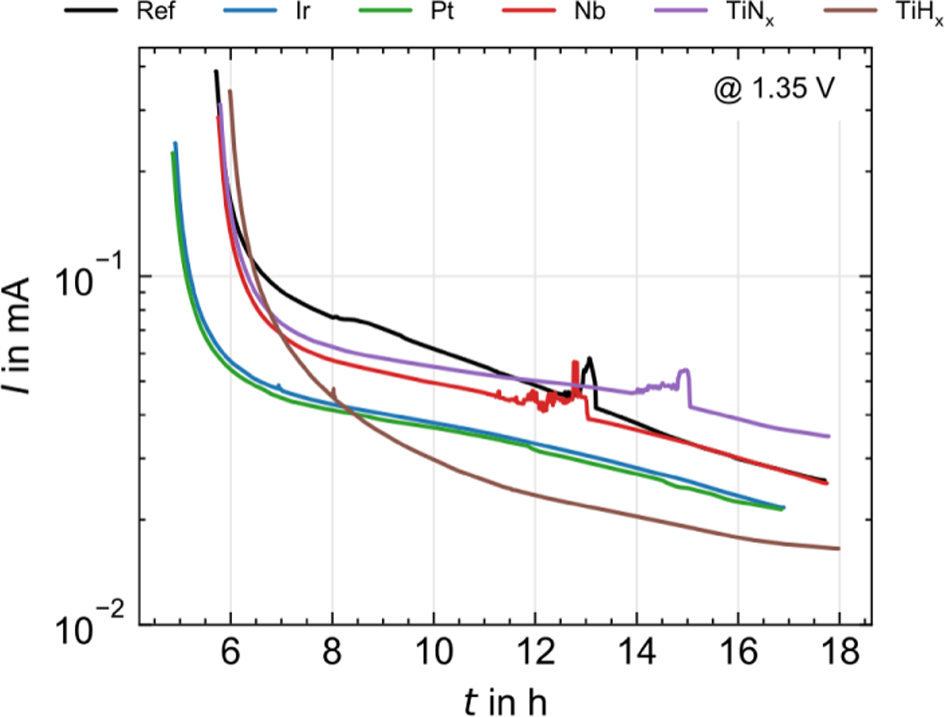
Corrosion current over time at 1.35 V in 0.5 mol L^–1^ H_2_SO_4_ at room temperature for all PTL variations in the ex situ rapid aging test.

Ex situ rapid aging tests are carried out to assess the corrosion stability of the coatings. Figure 5 shows the current *I* during a chronoamperometry (CA) measurement as a function of the exposure time to 0.5 mol L^–1^ H_2_SO_4_. The measured current indicates how strongly the corrosion reaction on the measured PTL occurs at the interface toward the electrolyte. In this case, a higher current indicates a stronger corrosion reaction. Some measurement curves show noise in the signal caused by unknown interference signals when recording the measurement data on the potentiostat. It was digitally reduced during data evaluation.

For all measured samples, the current drops sharply within a few hours at the start of the CA measurement but falls less as the exposure time progresses. The corrosion reaction thus slowly approaches an equilibrium state, which is not reached at the end of the test. Nevertheless, a trend toward corrosion stability can be derived from the level of the corrosion current at the end of the exposure time. The PGM coated and TiH_*x*_ PTLs show a significantly lower corrosion current than the reference, with Pt and Ir behaving almost identically. The different shape of the TiH_*x*_ curve could be related to the HCl induced structuring inside the pores at shaded Ti fibers, which causes a significantly different contact with the electrolyte compared to the directionally sputtered samples. The Nb coated PTL performs similarly to Ref, while the TiN_*x*_ coated PTL shows a higher corrosion current. In the literature, the corrosion resistance of TiN_*x*_ is strongly dependent on the manufacturing process.^27−29^

In the long term measurements in the electrolysis cell, qualitatively similar behavior is observed: Worsened stability for TiN_*x*_, Ref and Nb comparable, TiH_*x*_, Pt and Ir optimized. The ex situ rapid aging test is thus able to determine significant trends in corrosion stability ex situ. It should be added that the corrosion currents are not yet in equilibrium at the end of the test and that the conditions in the cell, particularly in the pore space of the PTL, are different regarding pH. Further investigation is needed for a quantitatively meaningful ex situ rapid aging test.

Subsequently, GIXRD measurements are carried out (Figure S3) to investigate the degradation effects of the treated PTL surface through the ex situ rapid aging test. As a result of the ex situ rapid aging tests, neither one of the coatings nor the TiH_*x*_ dissolved or changed chemically so that a change could be detected using GIXRD. This finding contradicts the dissolution of the Nb layer observed in the long term measurements, which will be discussed later.

#### Reproducibility and Contact Resistance Measurements

In order to demonstrate a reproducible improvement in CL contacting, short-term measurements (approximately 24 h) are carried out three times for each PTL variation in the same cell with new material, supported by ex situ contact resistance measurements.

**Figure 6:**
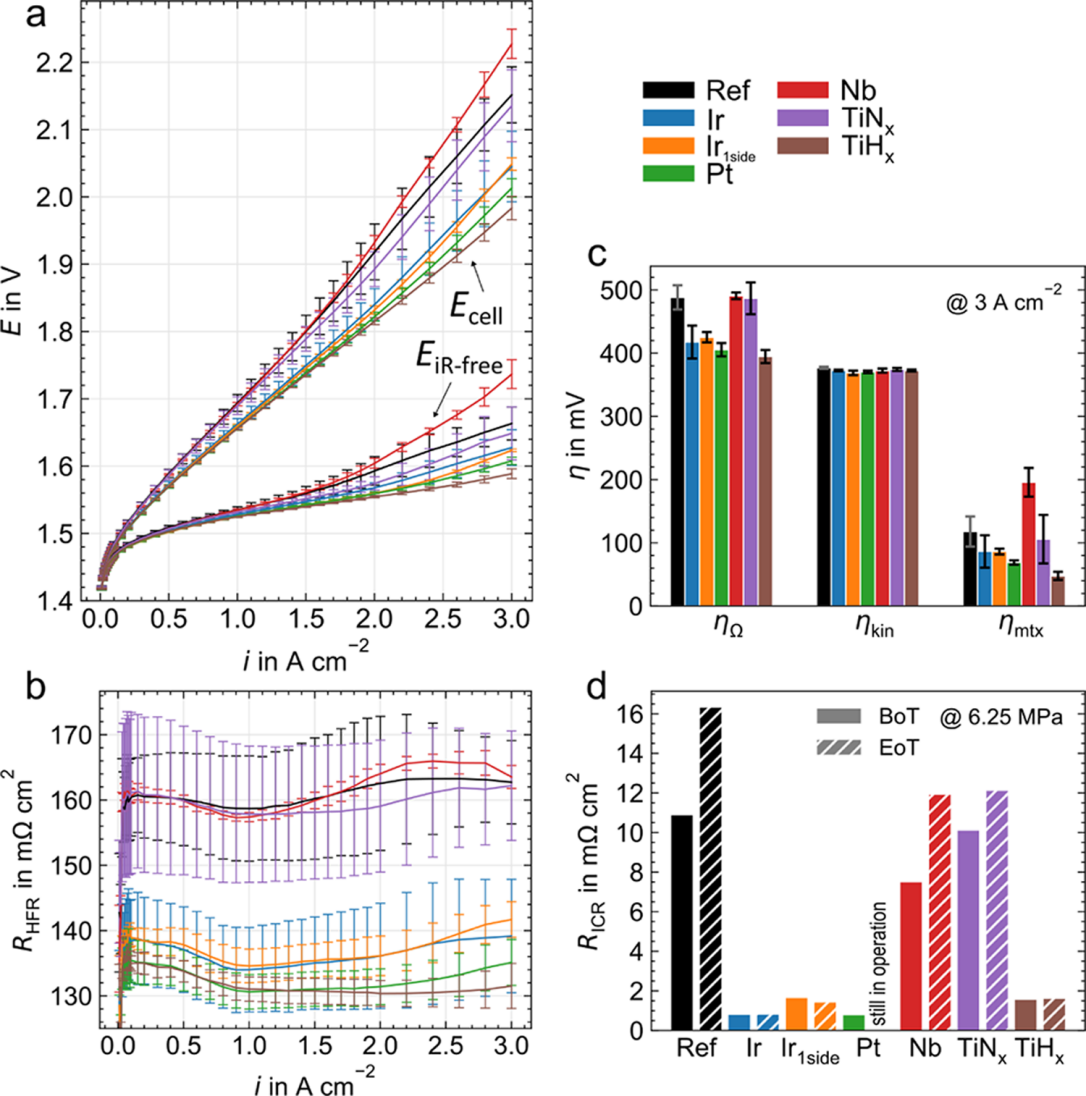
Three times repeated reproducibility measurements for all PTL coating variations: cell voltage and HFR-free voltage over current density (a), HFR (b), and overview (c) for ohmic (η_Ω_), kinetic (η_kin_), and mass transport plus residual overvoltages (η_mtx_). The measurements were conducted at 80 °C, ambient pressure, and with a Nafion 115 based CCM. (d) Ex situ contact resistance measurements begin (BoT, after 24 h) and end of test (EoT, after 2000 h) at 6.25 MPa.

Figure 6 shows the averaged polarization curves as well as HFR-free voltage (a), the HFR curves (b), and an overview of the overvoltages at a current density of 3 A cm^–2^ (c) for all samples including error bars.

The PGM coatings (Ir, Ir_1side_, Pt) significantly and reproducibly reduce the cell voltage compared to Ref, due to smaller HFRs and corresponding ohmic overvoltages. In addition, mass transport plus residual overvoltages are also lower, probably because a more homogeneous catalyst utilization reduces mass transport and ionic overvoltages in the CL. The comparison of Ir and Pt shows no significant difference in performance. In addition, the measurement of Ir_1side_ confirms that the interface toward the flow field has no measurable influence on the HFR.

The PGM-free coatings show different trends. TiN_*x*_ performs and contacts similarly to Ref here; this comparison differs from the long-term BoT polarization curves at 150 h, where TiN_*x*_ already appears to contain initial degradation. Nb also shows no performance improvement but similar HFR values and slightly increased mass transport plus residual overvoltages, the latter of which cannot be clearly explained and is not observed in the long term measurements. This performance deviates from the literature, where a powder-based Nb coating on a stainless steel PTL outperforms the reference sample.^16^ TiH_*x*_ shows the lowest cell voltage of all due to, on the one hand, the optimized HFR because of the high conductivity of the coating and, on the other hand, the increased contact area resulting from the surface structure.^14,15^ In addition, transport losses (mass, charge) are lowered, presumably due to a very homogeneous current distribution in combination with a gas removal enhancing surface structure.

The kinetic overvoltages of the samples do not differ much, as the same CL is used. Concerning the HFR, an unusual current density dependence is observed, which can possibly be explained by production-related impurities from the CL, which are not yet completely rinsed out of the CL after 20 h conditioning. This behavior is also observed in other tests with material from this manufacturer depending on the batch, but no longer occurs in the BoT HFR curves of the long-term measurement after 150 h. In addition, there are fluctuations between the widths of the error bars at 3 A cm^–2^ (9–53 mV or 1–9 mΩ cm^2^). The most important influence on this is CL inhomogeneity in connection with selecting the CCMs. The CCMs are provided in 25 cm^2^ sheets, from which four 4 cm^2^ samples are cut. If all three reproducibility measurements feature the same CCM sheet (e.g., for TiH_*x*_), the variation in performance is small. On the other hand, the reproducibility between different sheets is sometimes very poor (visible in Ir, for example). It is, therefore, assumed that the CCM accounts for a large part of the error bar and that the coatings perform very similar in all repetitions. A more reproducible CCM system could lead to much smaller variations within the sample repetitions.

Figure 6d presents the results of the contact resistance measurements as a bar plot at 6.25 MPa—the contact pressure of the electrolysis cell. All measured pressures from 0.125 to 10 MPa are plotted in Figure S4. A relative error of about 16% is calculated from three Ref samples. PTLs from the reproducibility measurement are used here as BoT samples, while EoT (shaded) are the long-term samples after the test. The Pt measurement cannot be measured EoT as it is still in operation.

Due to the formation of TiO_*x*_, the contact resistance of Ref worsens significantly after 2000 h. A strongly improved BoT contact resistance is measured for the PGM.^7,11,23^ In case of Ir, the conductivity remains the same after the long-term test, considering the error tolerance. The EoT Ir_1side_ sample also still has excellent contact, which shows that the interface toward the flow field does not appear to be passivated measurably. In line with the results of the HFR analysis, TiH_*x*_ shows a significant initial improvement in the contact resistance, which is still detected EoT and indicates good corrosion stability of the sample. However, a difference in the setup with the universal testing machine compared to the other samples cannot be ruled out for this sample due to the varying surface structure. Nb initially shows a slight improvement in the contact resistance with values comparable to Stiber et al.^16^ before a significant deterioration occurs during the long-term test. TiN_*x*_ also degrades during the test, and the comparison with similar tests^27,29^ shows that the manufacturing process of TiN_*x*_ has a decisive influence on its stability and contact resistance.

It should be noted that the rigid, gold-plated pressure plate of the universal testing machine contacts and compresses differently than the CL in the cell. Nevertheless, the overall trends match the ohmic overvoltages of the reproducibility measurements very well, so these can be attributed to the varied contact resistance to the CL.

#### Overvoltage Separation of Degradation Rates

**Figure 7:**
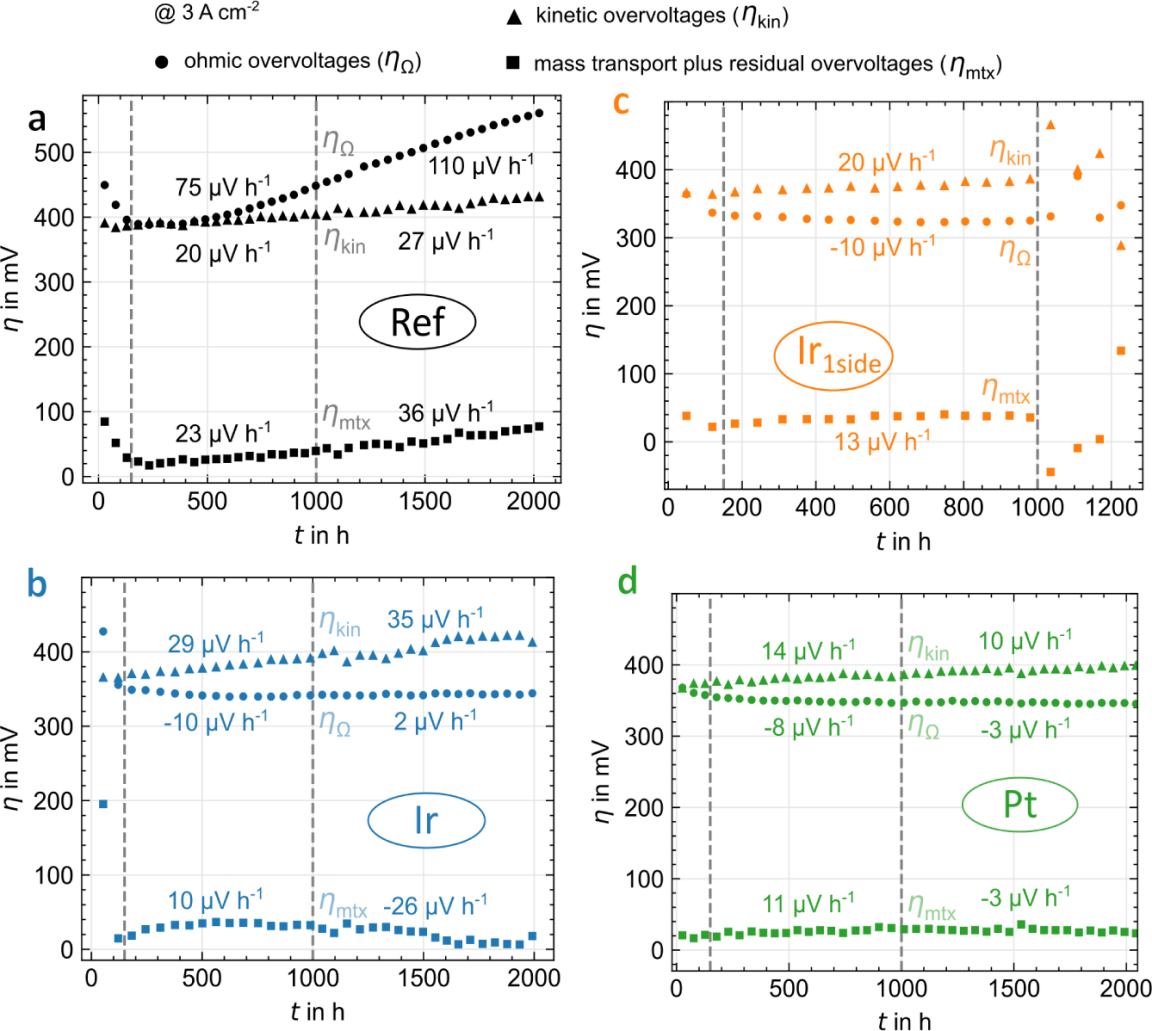
Ohmic (circles), kinetic (triangles), and mass transport plus residual (squares) overvoltages at 3 A cm^–2^ over time for Ref (a) and PGM (b–d) PTL coatings including partial degradation rates for both halves of the long-term test, derived from polarization curves at 3 A cm^–2^.

**Figure 8:**
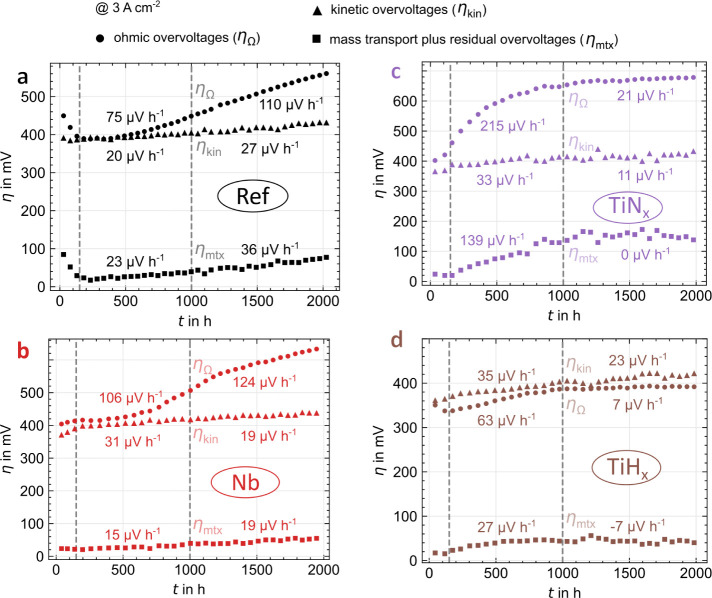
Ohmic (circles), kinetic (triangles), and mass transport plus residual (squares) overvoltages at 3 A cm^–2^ over time for Ref (a) and PGM-free (b–d) PTL coatings including partial degradation rates for both halves of the long-term test, derived from polarization curves at 3 A cm^–2^.

The degradation rate is divided into partial rates corresponding to the three overvoltages using a voltage breakdown of the measured polarization curves to investigate the origin of degradation. Figures 7 and 8 present the ohmic, kinetic, and mass transport plus residual overvoltages over time, including the partial degradation rates for both halves of the test. The 3 A cm^–2^ steps from the third polarization curves of the characterization phases are selected according to stress phases. The sum of the partial degradation rates deviates only <1 μV h^–1^ from the total rate in Figure 4.

The *ohmic overvoltages* (circles in Figures 7 and 8) dominate the degradation for Ref and the PGM-free coatings, especially in the first half of the test with partial rates between 63 μV h^–1^ (TiH_*x*_) and 215 μV h^–1^ (TiN_*x*_). Ref and Nb (Figure 8a,b) degrade over the entire test duration, probably due to oxide formation. For TiN_*x*_ and TiH_*x*_ (Figure 8c,d), the degradation decreases significantly in the second half of the test, and a saturation effect occurs, like in the overall degradation rate. The partial degradation rate in the second half is 7 μV h^–1^ for TiH_*x*_; overall, TiH_*x*_ reduces the ohmic partial degradation rate by 62% compared to Ref. The curve suggests a diffusion-driven oxidation of the material, as the time dependence appears square-root shaped.^30^ In addition, the layers are exposed to less oxygen in the second half of the test, particularly in the stress phases, as less current flows at the applied 2 V with increased degradation. The ohmic overvoltages of the PGM coatings (Figure 7) show negative partial degradation rates, especially in the first half of the test (−8 to −10 μV h^–1^), i.e., an improved contact over time, as reported elsewhere.^2,10,12^ Apparently, the passivation of the PTL is not only prevented, but a slow creeping adjustment of the CL also improves the contact.^31^ This effect probably also occurs with PGM-free coatings but is overlaid by the passivation there. Another influence that can reduce the ohmic overvoltages over time is membrane thinning.^32,33^ Even in the second half of the measurement, no strong deterioration is measurable (partial rates of 2 and −3 μV h^–1^ for Ir and Pt, respectively). An alternative representation of these findings is shown in Figure S2 as HFR values over time.

The *kinetic overvoltages* (triangles in Figures 7 and 8) for all PTL variations are about 400 mV with partial degradation rates between 10 and 35 μV h^–1^. Thus, they dominate the overall degradation rate for the PGM coatings (Figure 7). The scattering and strength of the degradation are related to the CCM used here and can vary for other manufacturers. Interestingly, the development of the kinetic overvoltages here does not depend on the applied coating or the test duration. Notably, inaccuracies in the Tafel regression, propagation of EIS errors, and mass transport plus residual overvoltages at low current densities can distort the kinetic overvoltages. In addition, there is a variation between the samples, which was already attributed to homogeneity fluctuations of the CCM when evaluating the reproducibility measurements. Dissolution^11^ or agglomeration^2,34^ is a possible cause of catalyst degradation, which reduces the electrochemical surface area (ECSA) and thus increases the overvoltage. In addition, impurities in the feedwater^35,36^ or dissolved Ti^2^ can contaminate the CL.

The *mass transport plus residual overvoltages* (squares in Figures 7 and 8) show a trend analogous to the ohmic overvoltages for all coating variations. The partial degradation rates are lower for the PGM coatings than the PGM-free ones due to the more homogeneous mass transport and less ionic overvoltages in the CL associated with improved contact. It is striking (and difficult to explain) that the partial rates for the PGM coatings are negative in the second half of the test (−26 and −3 μV h^–1^ for Ir and Pt, respectively). For the PGM-free coatings, the overvoltages for Ref and Nb increase slightly in the second half, while the saturation effect already described occurs for TiH_*x*_ and TiN_*x*_. Apart from the mass transport, the overvoltages surely contain other unknown effects, especially in the first half of the test for TiN_*x*_ (partial rate 139 μV h^–1^. A connection to the catalyst layer resistance is imaginable and could be linked to the ohmic overvoltages.^12,37^ Generally, the mass transport plus residual overvoltages are at the end of the breakdown and show the most significant inaccuracy.

#### Reversible Degradation

After investigating irreversible degradation effects, this section examines the reversible components of the various overvoltages and their dependence on the coating of the PTL.

**Figure 9:**
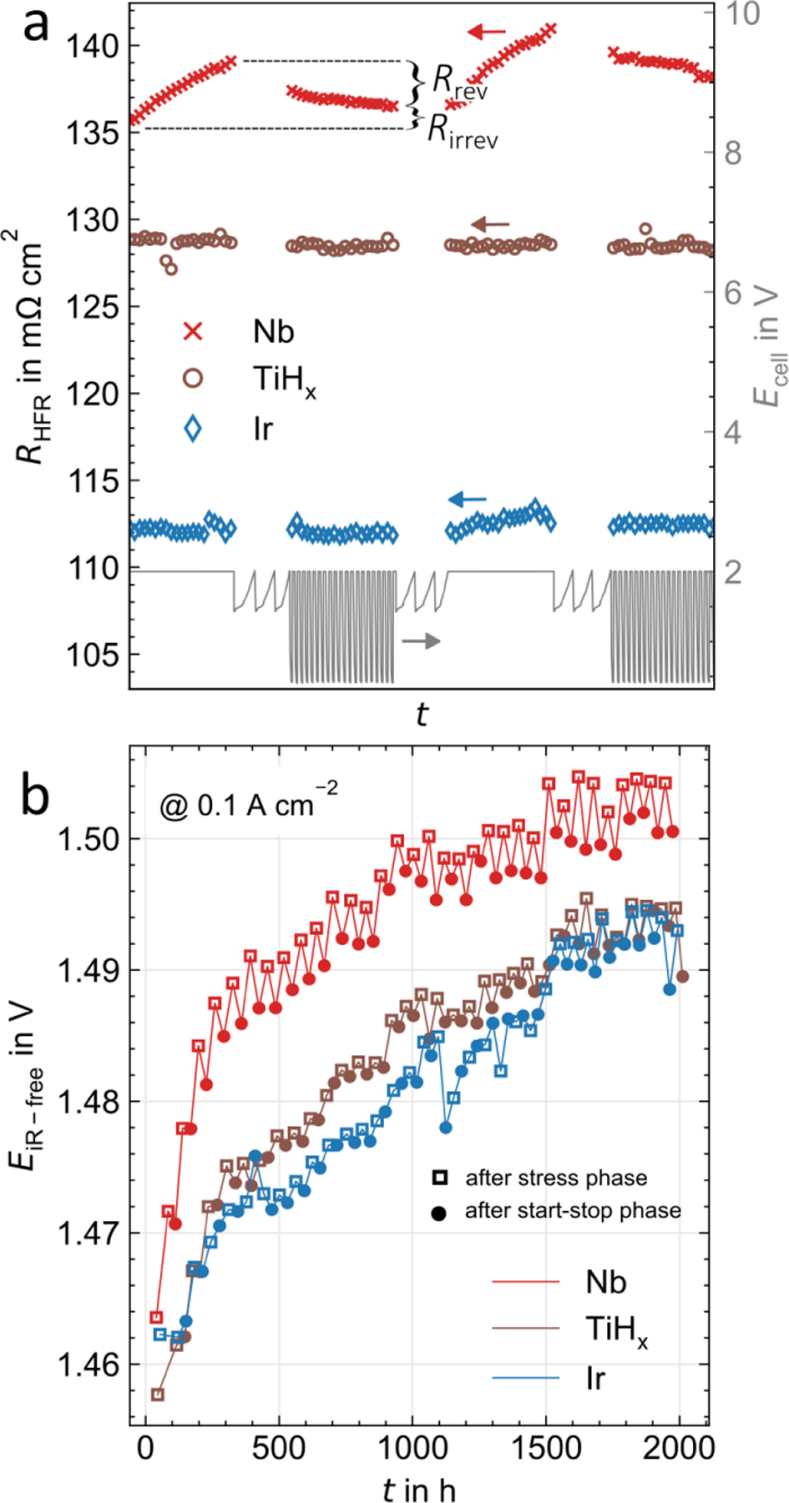
Reversible degradation effects for Nb (red), TiH_*x*_ (brown), and Ir (blue)—all other plots can be found in the Supporting Information. (a) Ohmic reversible (*R*_rev_) and irreversible (*R*_irrev_) degradation in a roughly 120 h wide cutout (values digitally synchronized for clarity) of HFR values over time. The corresponding protocol phases (stress phase, characterization, start–stop phase, and second characterization) as visualized by the cell voltage in the lower part, while no HFR values are plotted for the characterization curves. (b) Kinetic degradation displayed as the HFR-free voltage at 0.1 A cm^–2^ extracted from polarization curves over the full testing time after stress (squares) and start–stop phases (circles).

To assess the *reversible ohmic overvoltages*, Figure 9a shows a selection of the HFR measurements for Nb (red crosses), TiH_*x*_ (brown circles) and Ir (blue diamonds) during stress and start–stop phases. The total cell voltage is shown in gray on the second *y*-axis. For Nb, a continuous increase in resistance can be observed in the stress phase at 2 V, which is recovered in the start–stop phase (2 V and OCV alternating in 30 min intervals) by the proportion *R*_rev_, while an irreversible proportion remains (*R*_irrev_). *R*_rev_ is in the range of a few mΩ cm^2^ for Ref and Nb and up to 10 mΩ cm^2^ for TiN_*x*_, while the effect cannot be observed for the PGM coatings (Pt, Ir, Ir_1side_) and TiH_*x*_ at all. Cut-outs from the HFR curves for all coatings are shown in Figures S5–S7. It is assumed that poorly conductive TiO_*x*_ detaches from the oxidized Ref or TiN_*x*_ interface due to the changing potential,^13,38^ which is also probable for Nb and NbO_*x*_.^39^ This would lead to a temporarily improved electrical contact before the next stress phase restores the oxide layer. Alternatively, a chemical reduction of TiO_*x*_ or NbO_*x*_ to more conductive Ti or Nb is conceivable. Both effects do not occur for Pt and Ir due to their significantly lower tendency to oxidation. For TiH_*x*_, the bond between Ti and H appears to be strong enough to prevent reversible oxidation or dissolution.

The *reversible kinetic overvoltages* are shown in Figure 9b exemplary for Nb, TiH_*x*_ and Ir as HFR-free voltage at 0.1 A cm^–2^ from the third polarization curves of each characterization (after stress phase depicted as a square, after start–stop phase as a point). Since the ohmic overvoltages are subtracted from the total voltage and the mass transport plus residual overvoltages are usually negligible at low current densities, it is assumed that the changes over time are due to changes in the kinetic overvoltages. In addition to an increasing irreversible trend, a recovery of the kinetics is observed after the start–stop phase. It is assumed that the potential change causes a conversion of the CL material to a catalytically more active Ir configuration.^40^ As a catalyst-specific effect and thus primarily independent of the coating, it occurs for all measured samples (see Figures S5–S7). However, the amplitude of the recovered voltage is lower for PGM and TiH_*x*_ (about 1 mV) compared to Ref, Nb, and TiN_*x*_ (several mV). An electrically sufficient and homogeneous contact of the CL could reduce the short-term degradation of the catalyst in the stress phase. However, as previously stated, the choice of coating has no evident influence on irreversible catalyst degradation.

In evaluating the *mass transport plus residual overvoltages*, no apparent reversible effect can be determined from the polarization curves at 3 A cm^–2^ over time. The plots are shown in Figures S5–S7.

#### 5000 h Pt Coated PTL Long-Term Measurement

**Figure 10:**
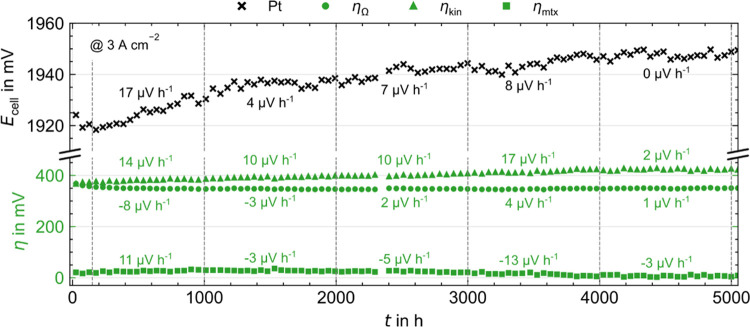
Cell voltage (black crosses) as well as ohmic (circles), kinetic (triangles), and mass transport plus residual overvoltages (squares) over time extracted from polarization curves after stress phases at 3 A cm^–2^ for the Pt long-term test with total and partial degradation rates in 1000 h intervals.

Different from the other samples, the Pt sample continues to be measured beyond 2000 h, and after 5000 h more data is saved and evaluated. Figure 10 shows the cell voltage as well as ohmic, kinetic, and mass transport plus residual overvoltages at 3 A cm^–2^ from the third polarization curves after stress phases, including the degradation rates and partial rates in 1000 h intervals. Overall, the increase in voltage is only about 30 mV, similar to Liu et al.^11^ After 17 μV h^–1^ in the first 1000 h, the total degradation rate drops to single-digit levels until 0 μV h^–1^ in the last interval. Degradation of the ohmic overvoltages (i.e., of the coating, green circles) is barely detectable even after 5000 h since the partial rates are only a few μV h^–1^. Over the entire time, the degradation of the kinetic overvoltages (triangles) makes up the majority of the rate. The mass transport plus residual overvoltages (squares) decrease continuously from 1000 h, whereby a connection to inaccuracies in the Tafel regression is suspected. Despite the EoT characterization not being conducted yet, the Pt coating does not seem to degrade significantly even after 5000 h.

#### EoT Characterization

**Figure 11:**
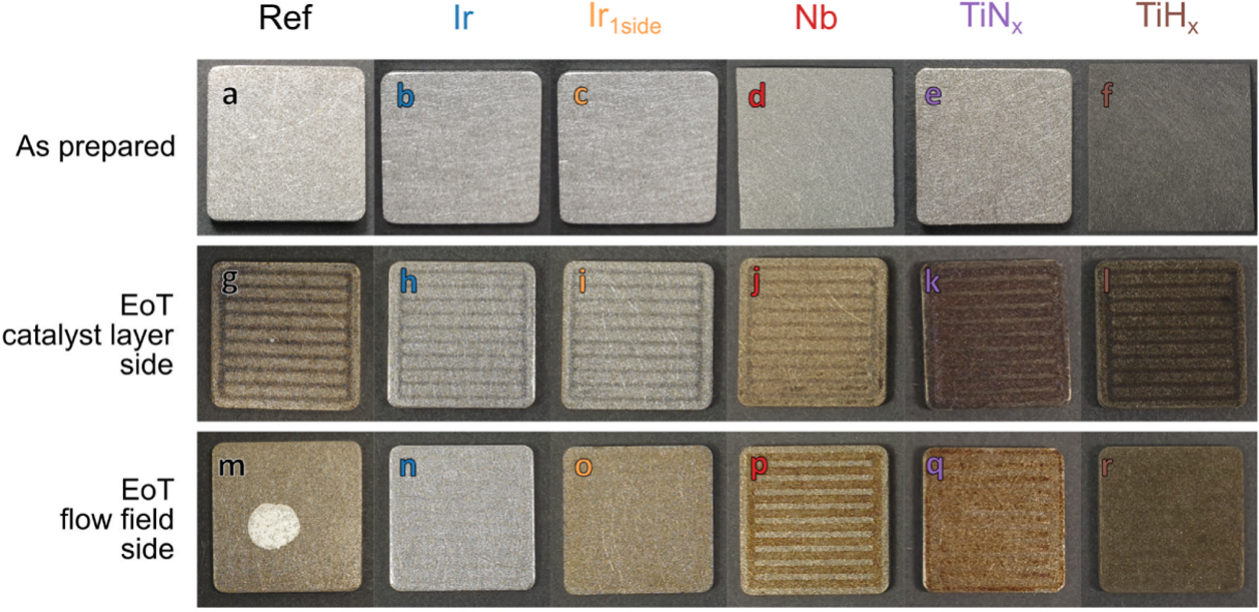
Photos of coated PTLs as prepared (a–f) and after long-term test on the CL side (g–l) and flow field side (m–r). Samples (d) and (f) are for corrosion measurements, which is why they do not have the rounded shape of the others. The white spot on the flow field side of Ref stems from a conductive adhesive for the ex situ end of test characterization.

The long-term PTLs are examined EoT with the naked eye and characterized in more detail concerning their degradation using GIXRD and FIB-SEM-EDX cross-section images. Figure 11 shows photos of the PTLs before measurement (“as prepared”) and both sides EoT. The Pt samples are not shown because the PTL is still being measured. The white spot on the Ref EoT PTL (m) stems from an adhesive for the FIB-SEM-EDX images. Initially, all coated samples are silver (a–e); only the TiH_*x*_ PTL (f) is darker due to the roughening etching step. The color does not change for the Ir samples (h,i,n) during the test, while a visible discoloration occurs on uncoated interfaces (g,m,o) and PGM-free coated PTLs (j–l, p–r). The discoloration is particularly strong for TiN_*x*_ (k,q), which showed the strongest degradation. The oxidation of the Ir_1side_ flow field side (o) does not significantly influence the cell performance or the contact resistance. All samples show dark stripes on the CL side, reflecting the flow field channels. In these areas, a redeposition of released CL material probably occurs, consistent with the comparatively strong degradation of the CCM in this setup. This finding contradicts the observations reported in the literature,^11,13^ where CL material stuck to the PTL under the lands of the flow field during cell disassembly. Another interesting observation is that the dark stripes are narrower for samples with good conductivity, such as Ir (h), and wider for those with poor conductivity, such as TiN_*x*_ (k). It is conceivable that an improved electrical connection of the CL material in the channel reduces the release. Finally, it is found that only for Nb a flow field structure is also recognizable on the flow field side (p), which may be due to redeposition of the dissolved Nb coating, which is determined in the following characterization.

**Figure 12:**
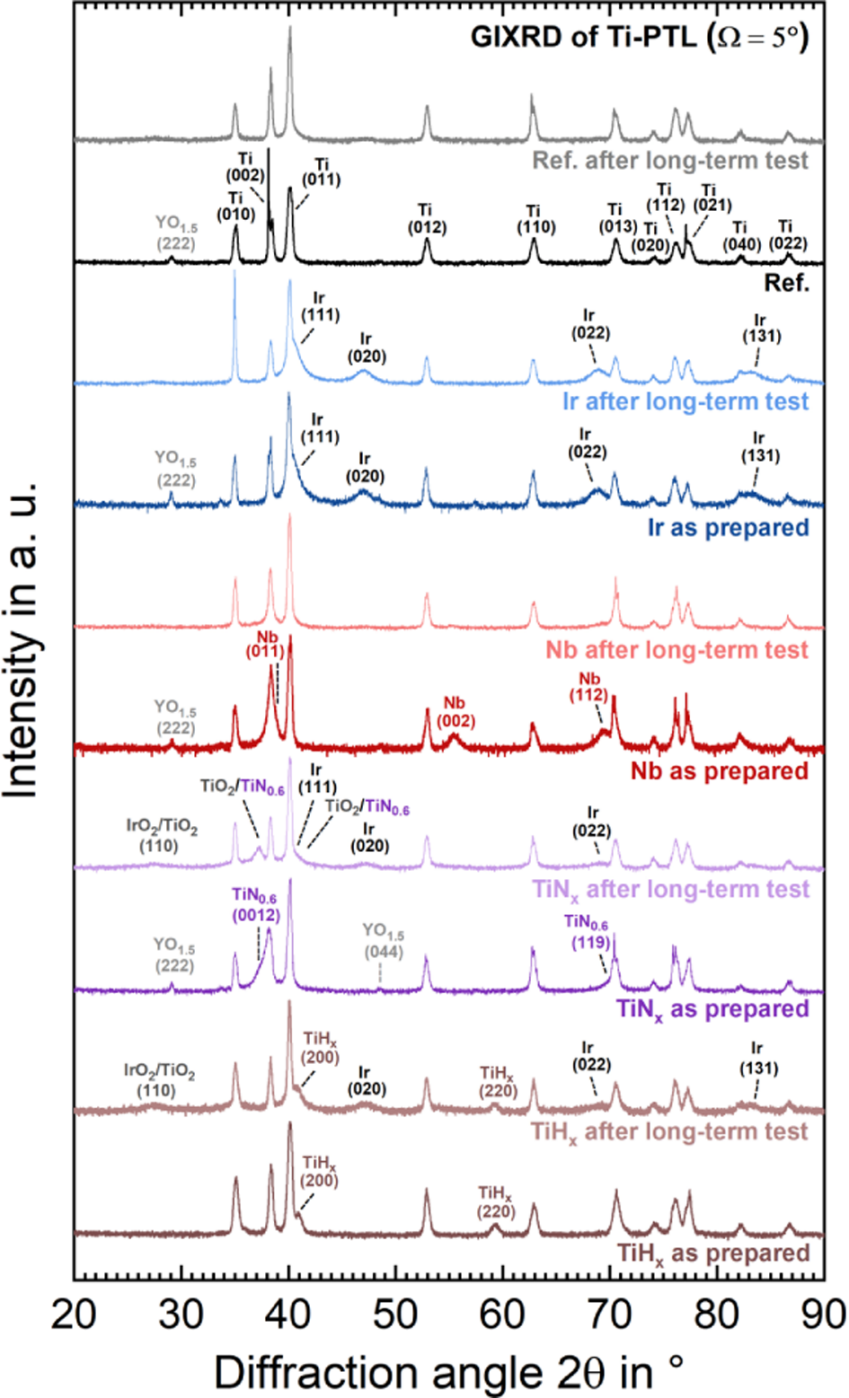
GIXRD spectra for PGM and PGM-free PTL coatings before and after the 2000 h long-term test.

**Figure 13:**
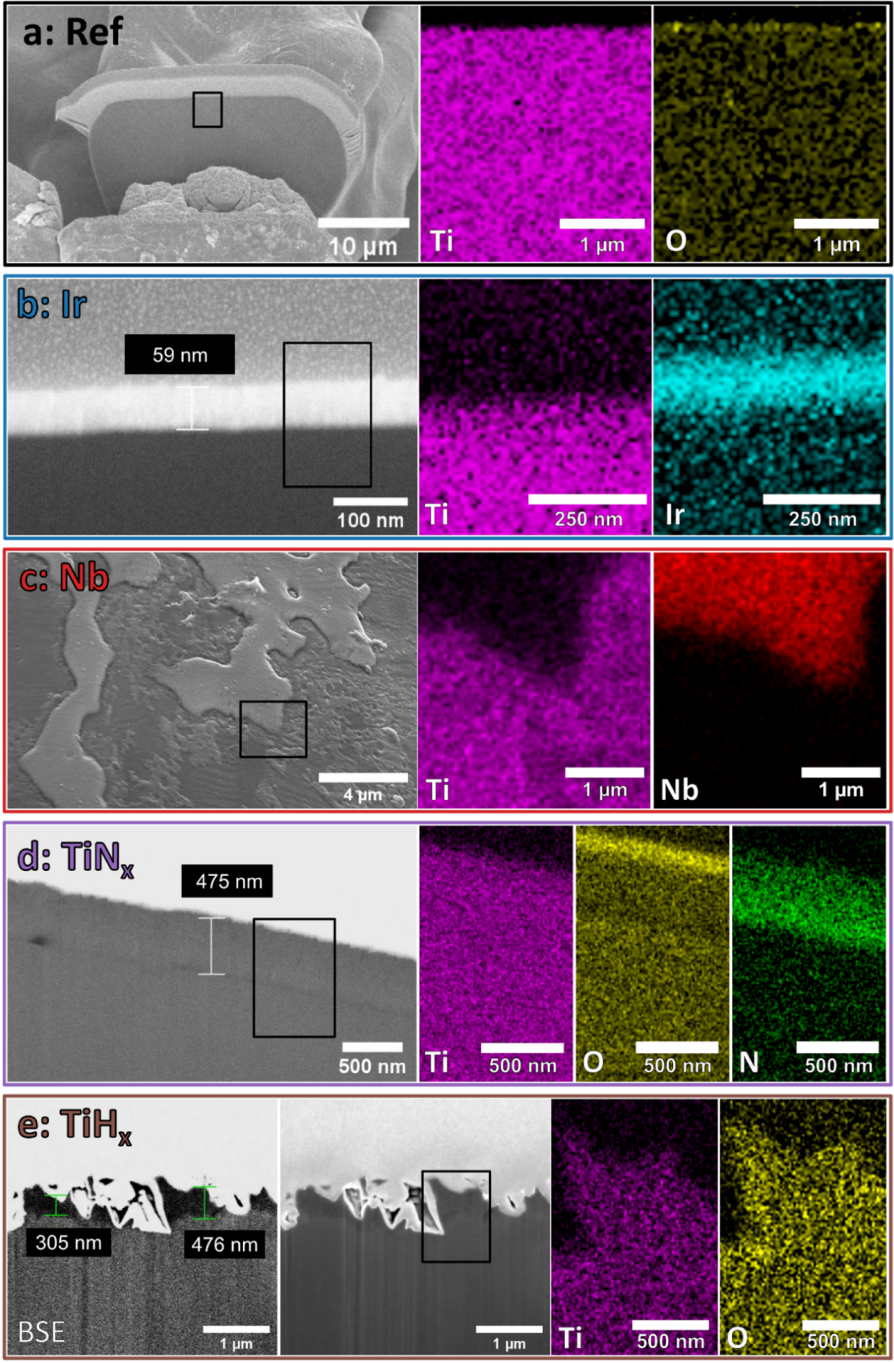
Long-term EoT degradation characterization of the PTL variations utilizing FIB-SEM images with EDX mapping of important elements located in black rectangles for Ref (a), Ir (b), Nb (c), TiN_*x*_ (d), and TiH_*x*_ (e). Since the Pt sample is still in operation, no data can be obtained. The yellow EDX oxygen signal in (a) is optimized in terms of contrast and brightness for clarity of the thin accumulation in the surface region.

Figure 12 presents the GIXRD measurements of the coated EoT PTLs, and Figure 13 the FIB-SEM cross-section images with relevant EDX maps of sections marked with a black box. For all samples, a thin Pt layer is deposited on the fiber to improve the cutting quality (see Experimental Section), which can be seen in the SEM image for Ref (Figure 13a), for example. Since the PTL starting material has a YO_*x*_ particle contamination, the reflections of the YO_1.5_ phase^41^ (COD database code: 1009013) are visible in the GIXRD spectrum.

For the TiN_*x*_ and TiH_*x*_ samples, the GIXRD spectrum after the long-term test in Figure 12 shows the broad and less intense (020) and (022) reflex of the nanocrystalline Ir phase^42^ (COD database code: 9008517), which appears at 2θ = 47.1° and 2θ = 69°. This finding implies that small amounts of the Ir catalyst material of the CCM can be found on the PTL after removal, which is consistent with the theory based on Figure 11.

For some samples, the GIXRD spectrum after the long-term test also shows a broad and low-intensity reflection of another nanocrystalline phase, which is most clearly visible at 2θ = 27.5°. Both the (110) main reflection of the rutile structure of IrO_2_^43^ (COD database code: 1538153) and TiO_2_^44^ (COD database code: 1530150) could have caused this reflection. Further investigations are needed to clarify this issue thoroughly.

For Ref, the GIXRD spectrum before the long-term test shows the typical reflections of the Ti phase.^42^ EoT TiO_*x*_ cannot be unquestionably detected using GIXRD, but the EDX mapping (Figure 13a) suggests a thin oxygen-rich layer on the surface of the Ti fiber (narrow yellow line).

The Ir coating displays unchanged X-ray reflections in the GIXRD spectrum in Figure 12 before and after the long-term test. An oxidic rutile structure is barely detectable, which underlines the chemical stability. The FIB-SEM images and the EDX mapping (Figure 13b) demonstrate that the layer thickness of about 59 nm (including about 12 nm Ti interlayer) after the test is unchanged from (55 ± 4) nm before the test. Overall, no dissolution or other layer degradation seem to take place, which explains the excellent stability in the long term measurement. This observed stability is consistent with that of Liu et al.,^11^ where an intact Ir layer with unchanged thickness was also detected after a long-term test (4000 h). However, the EDX spectrum identified a thin IrO_2_ layer on the outside, which in the case presented here cannot be determined with certainty due to the sample preparation.

Since the Pt coated PTL is still in operation, no cross-sectional investigation can be carried out. However, the electrolysis performance shows a lower degradation rate than the Ir coating and also ohmic degradation partial rates of ≤4 μV h^–1^ over 5000 h. This indicates comparable stability, which has also been observed in the literature over 4000 h.^11^ The result contradicts the observations of Rakousky et al.,^12^ who found an increased degradation rate of 63 μV h^–1^ in a 2000 h test for higher current densities (≥2.5 A cm^–2^), which has been associated with a detachment of the Pt coating. They attribute this effect to chemical corrosion and/or mechanical influences such as bubble formation or layer cracks. One difference in comparison to the presented study could be the different layer thickness (about 50 vs 200 nm) and the Ti interlayer added here. However, for a precise verification of the hypotheses, a cross-sectional study of the EoT coating is necessary.

The FIB-SEM image and the EDX mapping of the Nb-coated PTL (Figure 13c) reveal that the coating has partially either dissolved or detached. The soft contours of the coating residues indicate that the Nb layer dissolves during the electrolysis process. This process can also promote localized detachment of the Nb layer. The coarse column growth (compare Figure 2) may offer a good surface for an attack. In the GIXRD spectrum after the long-term test in Figure 12, the reflections of the Nb phase have also largely disappeared, which confirms this observation. In contrast, Nb can still be detected with GIXRD (not shown here) on the flow field side of the PTL, so that a corrosion reaction involving Nb may only take place at the interface between PTL and CCM. In the ex situ rapid aging test, no dissolution has been observed, either due to a short measurement time or the lack of comparability of the setups. In the literature, good stability of Nb in the electrolysis cell is also reported over a longer period, whereby the temperature (65 °C versus 80 °C), measurement duration (1000 h versus 2000 h), and especially the deposition methods differ (plasma-spraying with many μm thickness versus about 200 nm sputtering).^16^ The long-term stability of especially sputtered Nb coatings thus requires further investigations to determine the conditions for a successful application.

The FIB-SEM images of TiN_*x*_ (Figure 13d) display that the measured layer thickness after the test is approximately 475 nm, which is in the range of the previous layer thickness of (473 ± 11) nm. The EDX mapping reveals a significantly increased oxygen signal and a lower nitrogen signal in the edge region of the TiN_*x*_ layer, indicating the near-surface conversion of TiN_*x*_ to a comparably thick TiO_*x*_ layer.^28,29^ A slight peak shift can be observed in the GIXRD in Figure 12: a TiN_*x*_ shoulder reflection at 2θ = 37.5° before the test has become a single slightly shifted reflection after the test, which can be assigned to both the TiO_2_^44^ and the TiN_0.6_ phase^45^ (COD database code: 1100040), confirming the observation from the EDX mapping. This transformation probably occurs during the first 1000 h, before the diffusion-driven oxidation reaches a saturated state and the degradation rate significantly decreases, as observed in the performance evaluation. Interestingly, this effect is not observed for Ref, probably due to the roughened surface of TiN_*x*_ that allows a better attack of the oxygen. The stability can be optimized in the future by a different manufacturing process (e.g., electrochemical^27^ or thermal^28^ TiN_*x*_), since excellent corrosion resistance has already been demonstrated in the literature.

In the FIB-SEM image (Figure 13e) after the long-term test, TiH_*x*_ still exhibits the characteristic spike-like surface structure originating from the etching step. The average height is still in the range of 300–500 nm. Dark spots in the BSE image mark areas with lower average atomic number, which indicates the presence of hydrogen even in deeper regions of the PTL after the test. The EDX mapping displays an oxygen signal within the spike-like structure but without increased accumulation on the surface as for Ref or TiN_*x*_. It is assumed that degradation occurs primarily in places with less TiH_*x*_ due to irregularities in the etching process or the structuring, so these sites become inactive in the first half of the test. After that, the degradation slows down considerably, and a saturation effect occurs since further TiH_*x*_ bonds only convert to TiO_*x*_ very slowly. An optimization of the HCl treatment could reduce this effect and increase the corrosion resistance of TiH_*x*_. In the before and after GIXRD spectra in Figure 12, no significant changes in the X-ray reflections of the TiH_*x*_ phase are visible, which means that TiH_*x*_ is still present after 2000 h. TiH_*x*_ has also been detected in the literature by XRD after 100 h^14^ and 500 h tests.^15^

Overall, the PGM-free coatings lag behind the PGM coatings in two aspects. The first is an initial increase in the contact resistance between PTL and CL compared to PGM coatings, which is not performance-limiting for TiH_*x*_ in short-term tests. Nb and TiN_*x*_, however, are behind Ir and Pt in terms of conductivity, which causes a direct increase in ohmic overvoltages. The second aspect is long-term stability. While the PGM coating (especially for Ir) shows no significant signs of degradation, Nb dissolves over time in the corrosive environment. TiN_*x*_ further deteriorates in terms of conductivity due to the incorporation of oxygen, which replaces the nitrogen and/or binds to the titanium. The latter process is probably also present for the TiH_*x*_ sample to a lesser extent. Further development of the PGM-free coating methods based on the literature discussed in the individual paragraphs could reduce these problems, making it possible to replace PGM with PGM-free coatings. Furthermore, the use of PGM-free coatings could be an option at low current densities and consequently less corrosive environments. Elevated current densities and very low catalyst loadings (and the resulting low in-plane conductivity of the catalyst layer) currently represent limitations of PGM-free coatings.

## Summary and Conclusion

Long-term stability and excellent contact resistance with minimal overvoltages due to passivation are crucial for PEM electrolyzers. This study extensively compares seven PTL configurations in situ and ex situ in short and long-term tests supported by ex situ rapid aging and contact resistance measurements. It should be noted that the findings may not be generalizable, but above all, they are significant for the test protocol used here. PGM coatings (Pt and Ir) significantly improve contact resistance and effectively prevent an increase in ohmic overvoltages in the cell. For Ir, there is no detectable structural change in the layer after 2000 h. The effect of Ir also remains if the contact surface toward the flow field remains uncoated. Pt shows a comparable performance to Ir and protects effectively even for 5000 h with an overall degradation rate of 7 μV h^–1^.

The PGM-free coatings behave differently: Nb exhibits no improvement in contact resistance and an overall degradation slightly stronger than Ref while dissolving during full-cell operation. TiN_*x*_ provides no initial change in the contact resistance but ages quickly due to strong oxidation of the layer in the first 1000 h of the long-term measurement (degradation is 43% greater than for Ref overall). TiH_*x*_ produced by an HCl etching step initially improves the contact resistance comparable to PGM coatings, then degrades similarly to TiN_*x*_ with a saturation effect but remains significantly better than the uncoated PTL. TiH_*x*_ is, therefore, the best PGM-free variant, with a degradation reduction of 49% overall and 62% in ohmic overvoltages compared to Ref.

The ex situ rapid aging tests qualitatively align with the trends of the long-term tests but without quantitative significance or degradation effects such as the dissolution of Nb.

Dividing the degradation rates into partial rates allows more profound insight into the cell degradation over time: PGM coatings even exhibit improvements in ohmic and mass transport plus residual overvoltages over time, while the degradation of the CL (kinetic overvoltages) dominates the degradation rate. The PGM-free coatings demonstrate a correlation between the overall degradation and the ohmic overvoltages, usually accounting for the largest share of the total degradation rate. The method could be routinely integrated into long-term measurements to understand degradation better and make more precise statements about degradation effects. In addition, reversible degradation is observed as an improvement in performance after start–stop phases, which occurs for all samples in kinetic overvoltages and in ohmic overvoltages for Ref, Nb, and TiN_*x*_. Future investigations should examine the use of single-side coated PTLs and even thinner PGM coatings over many thousands of hours with low loading catalyst layers, also in larger cells and stack systems. With regard to PGM-free coatings, the manufacturing process for TiN_*x*_ must be optimized and e.g., Nb compounds should be used instead of pure metal to enable better stability in the cell and comparable behavior with the PGM coatings.

## Supporting Information Available

Additional data on cross-section SEM images, HFR degradation including rates over time, GIXRD of ex situ rapid aging test, contact resistance measurements for all pressures, and long-term reversible and irreversible overvoltages for all PTL variations (PDF)

## Abbreviations

BoT: begin of test
CA: chronoamperometry
CCM: catalyst coated membrane
CL: catalyst layer
COD: Crystallography Open Database
EDX: energy-dispersive X-ray spectroscopy
EIS: electrochemical impedance spectroscopy
EoT: end of test
FIB: focused ion beam
GDL: gas diffusion layer
GIXRD: grazing incidence X-ray diffractometry
HFR: high frequency resistance
LSV: linear sweep voltammetry
OCV: open circuit voltage
PGM: platinum group metal
PTL: porous transport layer
RHE: reversible hydrogen electrode
SEM: scanning electron microscope

## References

[ref1] Qiu C.; Xu Z.; Chen F.-Y.; Wang H. Anode Engineering for Proton Exchange Membrane Water Electrolyzers. ACS Catal. 2024, 14 (2), 921–954. 10.1021/acscatal.3c05162.

[ref2] Rakousky C.; Reimer U.; Wippermann K.; Carmo M.; Lueke W.; Stolten D. An Analysis of Degradation Phenomena in Polymer Electrolyte Membrane Water Electrolysis. J. Power Sources 2016, 326, 120–128. 10.1016/j.jpowsour.2016.06.082.

[ref3] Prestat M. Corrosion of Structural Components of Proton Exchange Membrane Water Electrolyzer Anodes: A Review. J. Power Sources 2023, 556, 232469 10.1016/j.jpowsour.2022.232469.

[ref4] Yuan X.-Z.; Shaigan N.; Song C.; Aujla M.; Neburchilov V.; Kwan J. T. H.; Wilkinson D. P.; Bazylak A.; Fatih K. The Porous Transport Layer in Proton Exchange Membrane Water Electrolysis: Perspectives on a Complex Component. Sustainable Energy Fuels 2022, 6 (8), 1824–1853. 10.1039/D2SE00260D.

[ref5] Bautkinova T.; Prokop M.; Bystron T.; Bouzek K. Interface Between Anode Porous Transport Layer and Catalyst Layer: A Key to Efficient, Stable and Competitive Proton Exchange Membrane Water Electrolysis. Curr. Opin. Electrochem. 2025, 49, 101624 10.1016/j.coelec.2024.101624.

[ref6] Tao Y.; Wu M.; Hu M.; Xu X.; Abdullah M. I.; Shao J.; Wang H. High-Performance Porous Transport Layers for Proton Exchange Membrane Water Electrolyzers. SusMat 2024, 4, e230 10.1002/sus2.230.

[ref7] Liu C.; Carmo M.; Bender G.; Everwand A.; Lickert T.; Young J. L.; Smolinka T.; Stolten D.; Lehnert W. Performance Enhancement of PEM Electrolyzers Through Iridium-Coated Titanium Porous Transport Layers. Electrochem. Commun. 2018, 97, 96–99. 10.1016/j.elecom.2018.10.021.

[ref8] Liu C.; Wrubel J. A.; Padgett E.; Bender G. Impacts of PTL Coating Gaps on Cell Performance for PEM Water Electrolyzer. Appl. Energy 2024, 356, 122274 10.1016/j.apenergy.2023.122274.

[ref9] Mo J.; Steen S.; Han B.; Kang Z.; Terekhov A.; Zhang F.-Y.; Retterer S. T.; Cullen D. A.; Investigation of Titanium Felt Transport Parameters for Energy Storage and Hydrogen/Oxygen Production. In 13th International Energy Conversion Engineering Conference; American Institute of Aeronautics and Astronautics: Reston, Virginia, 2015. 10.2514/6.2015-3914.

[ref10] Kang Z.; Mo J.; Yang G.; Li Y.; Talley D. A.; Retterer S. T.; Cullen D. A.; Toops T. J.; Brady M. P.; Bender G.; Pivovar B. S.; Green J. B.; Zhang F.-Y. Thin Film Surface Modifications of Thin/Tunable Liquid/Gas Diffusion Layers for High-Efficiency Proton Exchange Membrane Electrolyzer Cells. Appl. Energy 2017, 206, 983–990. 10.1016/j.apenergy.2017.09.004.

[ref11] Liu C.; Shviro M.; Gago A. S.; Zaccarine S. F.; Bender G.; Gazdzicki P.; Morawietz T.; Biswas I.; Rasinski M.; Everwand A.; Schierholz R.; Pfeilsticker J.; Müller M.; Lopes P. P.; Eichel R.-A.; Pivovar B.; Pylypenko S.; Friedrich K. A.; Lehnert W.; Carmo M. Exploring the Interface of Skin-Layered Titanium Fibers for Electrochemical Water Splitting. Adv. Energy Mater. 2021, 11 (8), 2002926 10.1002/aenm.202002926.

[ref12] Rakousky C.; Keeley G. P.; Wippermann K.; Carmo M.; Stolten D. The Stability Challenge on the Pathway to High-Current-Density Polymer Electrolyte Membrane Water Electrolyzers. Electrochim. Acta 2018, 278, 324–331. 10.1016/j.electacta.2018.04.154.

[ref13] Srour T.; Kumar K.; Martin V.; Dubau L.; Maillard F.; Gilles B.; Dillet J.; Didierjean S.; Amoury B.; Le T. D.; Maranzana G. On the Contact Resistance Between the Anode and the Porous Transport Layer in a Proton Exchange Membrane Water Electrolyzer. Int. J. Hydrogen Energy 2024, 58, 351–361. 10.1016/j.ijhydene.2024.01.134.

[ref14] Bystron T.; Vesely M.; Paidar M.; Papakonstantinou G.; Sundmacher K.; Bensmann B.; Hanke-Rauschenbach R.; Bouzek K. Enhancing PEM Water Electrolysis Efficiency by Reducing the Extent of Ti Gas Diffusion Layer Passivation. J. Appl. Electrochem. 2018, 48 (6), 713–723. 10.1007/s10800-018-1174-6.

[ref15] Bautkinova T.; Utsch N.; Bystron T.; Lhotka M.; Kohoutkova M.; Shviro M.; Bouzek K. Introducing Titanium Hydride on Porous Transport Layer for More Energy Efficient Water Electrolysis with Proton Exchange Membrane. J. Power Sources 2023, 565, 232913 10.1016/j.jpowsour.2023.232913.

[ref16] Stiber S.; Sata N.; Morawietz T.; Ansar S. A.; Jahnke T.; Lee J. K.; Bazylak A.; Fallisch A.; Gago A. S.; Friedrich K. A. A High-Performance, Durable and Low-Cost Proton Exchange Membrane Electrolyser with Stainless Steel Components. Energy Environ. Sci. 2022, 15, 109–122. 10.1039/d1ee02112e.

[ref17] Daudt N. F.; Hackemüller F. J.; Bram M. Powder Metallurgical Production of 316L Stainless Steel/Niobium Composites for Proton Exchange Membrane Electrolysis Cells. Powder Metall. 2019, 62 (3), 176–185. 10.1080/00325899.2019.1607461.

[ref18] Wakayama H. Low-Cost Porous Transport Layers for Water Electrolysis Cells with Polymer Electrolyte Membranes. Mater. Res. Express 2024, 11 (8), 085501 10.1088/2053-1591/ad666e.

[ref19] Wang T.; Peng S.; Xu L.; Chen B.; Wang Y. TiCN-Coated Ti Felt for Enhanced Polymer Electrolyte Membrane Water Electrolysis. Ind. Eng. Chem. Res. 2025, 64 (1), 421–428. 10.1021/acs.iecr.4c03205.

[ref20] Gupta A.; Chellehbari Y. M.; Shahgaldi S. Achieving High Performance and Durability with Ultra-Low Precious Metal Nanolayer on Porous Transport Layer for PEMWE Application. J. Power Sources 2025, 630, 236088 10.1016/j.jpowsour.2024.236088.

[ref21] Ye H.; Chen L.; Shen D.; Li S.; Tu Z. Performance of Ta/TaN Coated Titanium Felt for Proton Exchange Membrane Water Electrolysis. Int. J. Hydrogen Energy 2024, 93, 1022–1030. 10.1016/j.ijhydene.2024.11.057.

[ref22] Gražulis S.; Daškevič A.; Merkys A.; Chateigner D.; Lutterotti L.; Quirós M.; Serebryanaya N. R.; Moeck P.; Downs R. T.; Le Bail A. Crystallography Open Database (COD): an Open-Access Collection of Crystal Structures and Platform for World-Wide Collaboration. Nucleic Acids Res. 2012, 40, D420–D427. 10.1093/nar/gkr900. 22070882 PMC3245043

[ref23] Liu C.; Wippermann K.; Rasinski M.; Suo Y.; Shviro M.; Carmo M.; Lehnert W. Constructing a Multifunctional Interface between Membrane and Porous Transport Layer for Water Electrolyzers. ACS Appl. Mater. Interfaces 2021, 13 (14), 16182–16196. 10.1021/acsami.0c20690. 33798332

[ref24] Becker H.; Dickinson E. J. F.; Lu X.; Bexell U.; Proch S.; Moffatt C.; Stenström M.; Smith G.; Hinds G. Assessing Potential Profiles in Water Electrolysers to Minimise Titanium Use. Energy Environ. Sci. 2022, 15 (6), 2508–2518. 10.1039/D2EE00876A.

[ref25] Suermann M.; Schmidt T. J.; Büchi F. N. Cell Performance Determining Parameters in High Pressure Water Electrolysis. Electrochim. Acta 2016, 211, 989–997. 10.1016/j.electacta.2016.06.120.

[ref26] Schuler T.; Schmidt T. J.; Büchi F. N. Polymer Electrolyte Water Electrolysis: Correlating Performance and Porous Transport Layer Structure: Part II. Electrochemical Performance Analysis. J. Electrochem. Soc. 2019, 166 (10), F555–F565. 10.1149/2.1241908jes.

[ref27] Liu Y.; Huang S.; Wang D.; Zhang H.; Shan D.; Peng S.; Shen G.; Wang L.; Wang X. Modifying Ti-Based Gas Diffusion Layer Passivation for Polymer Electrolyte Membrane Water Electrolysis via Electrochemical Nitridation. ACS Appl. Mater. Interfaces 2022, 14 (13), 15728–15735. 10.1021/acsami.1c22639. 35333508

[ref28] Toops T. J.; Brady M. P.; Zhang F.-Y.; Meyer H. M.; Ayers K.; Roemer A.; Dalton L. Evaluation of Nitrided Titanium Separator Plates for Proton Exchange Membrane Electrolyzer Cells. J. Power Sources 2014, 272, 954–960. 10.1016/j.jpowsour.2014.09.016.

[ref29] Takahashi K.; Kagawa T.; Tanaka K.; Kihira H.; Ushioda K. Reduction of Contact Resistance on Titanium Sheet Surfaces by Formation of Titanium Carbide and Nitride, and its Stability in Sulfuric Acid Aqueous Solution. ISIJ Int. 2019, 59 (9), 1621–1631. 10.2355/isijinternational.ISIJINT-2018-787.

[ref30] Unnam J.; Shenoy R. N.; Clark R. K. Oxidation of Commercial Purity Titanium. Oxid. Met. 1986, 26 (3–4), 231–252. 10.1007/BF00659186.

[ref31] Sadeghi Alavijeh A.; Khorasany R. M.; Habisch A.; Wang G. G.; Kjeang E. Creep Properties of Catalyst Coated Membranes for Polymer Electrolyte Fuel Cells. J. Power Sources 2015, 285, 16–28. 10.1016/j.jpowsour.2015.03.082.

[ref32] Rozain C.; Mayousse E.; Guillet N.; Millet P. Influence of Iridium Oxide Loadings on the Performance of PEM Water Electrolysis Cells: Part II – Advanced Oxygen Electrodes. Appl. Catal., B 2016, 182, 123–131. 10.1016/j.apcatb.2015.09.011.

[ref33] Siracusano S.; Trocino S.; Briguglio N.; Pantò F.; Aricò A. S. Analysis of Performance Degradation During Steady-State and Load-Thermal Cycles of Proton Exchange Membrane Water Electrolysis Cells. J. Power Sources 2020, 468, 228390 10.1016/j.jpowsour.2020.228390.

[ref34] Rakousky C.; Reimer U.; Wippermann K.; Kuhri S.; Carmo M.; Lueke W.; Stolten D. Polymer Electrolyte Membrane Water Electrolysis: Restraining Degradation in the Presence of Fluctuating Power. J. Power Sources 2017, 342, 38–47. 10.1016/j.jpowsour.2016.11.118.

[ref35] Sun S.; Shao Z.; Yu H.; Li G.; Yi B. Investigations on Degradation of the Long-Term Proton Exchange Membrane Water Electrolysis Stack. J. Power Sources 2014, 267, 515–520. 10.1016/j.jpowsour.2014.05.117.

[ref36] Millet P.; Ngameni R.; Grigoriev S. A.; Mbemba N.; Brisset F.; Ranjbari A.; Etiévant C. PEM Water Electrolyzers: From Electrocatalysis to Stack Development. Int. J. Hydrogen Energy 2010, 35 (10), 5043–5052. 10.1016/j.ijhydene.2009.09.015.

[ref37] Padgett E.; Bender G.; Haug A.; Lewinski K.; Sun F.; Yu H.; Cullen D. A.; Steinbach A. J.; Alia S. M. Catalyst Layer Resistance and Utilization in PEM Electrolysis. J. Electrochem. Soc. 2023, 170 (8), 084512 10.1149/1945-7111/acee25.

[ref38] Cherevko S.; Reier T.; Zeradjanin A. R.; Pawolek Z.; Strasser P.; Mayrhofer K. J. Stability of Nanostructured Iridium Oxide Electrocatalysts During Oxygen Evolution Reaction in Acidic Environment. Electrochem. Commun. 2014, 48, 81–85. 10.1016/j.elecom.2014.08.027.

[ref39] Chukwuike V. I.; Rajalakshmi K.; Barik R. C. Surface and Electrochemical Corrosion Analysis of Niobium Oxide Film Formed in Various Wet Media. Appl. Surf. Sci. Adv 2021, 4, 100079 10.1016/j.apsadv.2021.100079.

[ref40] Krenz T.; Rex A.; Helmers L.; Trinke P.; Bensmann B.; Hanke-Rauschenbach R. Reversible Degradation Phenomenon in PEMWE Cells: An Experimental and Modeling Study. J. Electrochem. Soc. 2024, 171 (12), 124501 10.1149/1945-7111/ad96e4.

[ref41] Baldinozzi G.; Bérar J. F.; Calvarin-Amiri G. Rietveld Refinement of Two-Phase Zr-Doped Y_2_O_3_. Mater. Sci. Forum 1998, 278–281, 680–685. 10.4028/www.scientific.net/MSF.278-281.680.

[ref42] Zemann J. Crystal structures, 2 nd edition. Vol. 1 by R. W. G. Wyckoff. Acta Crystallogr. 1965, 18 (1), 139 10.1107/S0365110X65000361.

[ref43] Goldschmidt V. M.; Barth T. F. W.; Lunde G.; Zachariasen W. H.; Geochemische Verteilungsgesetze der Elemente: 7. Die Gesetze der Krystallochemie. In Videnskapsselskapet (Kristiania)Matematisk-Naturvidenskapelig Klasse: Skrifter, 1926; I Kommission Hos Jacob Dybwad, 2007 Vol. 2, 37.

[ref44] Khitrova V. I.; Bundule M.; Pinsker Z. G. An Electron-Diffraction Investigation of Titanium Dioxide in Thin Films. Kristallografiya, 1977, (22), , 1253–1258.

[ref45] Lengauer W.; Ettmayer P. The Crystal Structure of a New Phase in the Titanium-Nitrogen System. J. Less-Common Met. 1986, 120 (1), 153–159. 10.1016/0022-5088(86)90637-5.

