# Supplementary material for: Degradation of PGM and PGM-free Coatings on PEMWE Porous Transport Layers
Source: ACS Appl Mater Interfaces. 2025 Mar 11;17(12):19070–85. doi: 10.1021/acsami.4c22455 (PMC11955940; doi:10.1021/acsami.4c22455)
Supplement: Supplementary file 1 — am4c22455_si_001.pdf [file am4c22455_si_001.pdf]

Supporting Information

Degradation of PGM and PGM-free Coatings on PEMWE Porous Transport Layers

Lukas Stein[1], Arne Dittrich[2,], Dominic C. Walter[2], Patrick Trinke[1], Boris Bensmann[1,*], Richard Hanke-Rauschenbach[1]*

[1]Leibniz University Hannover, Institute of Electric Power Systems, Appelstraße 9A, 30167 Hannover, Germany

[2]Institute for Solar Energy Research Hamelin (ISFH), Am Ohrberg 1, 31860 Emmerthal, Germany

*Corresponding authors

Boris Bensmann: boris.bensmann@ifes.uni-hannover.de; Arne Dittrich: dittrich@isfh.de

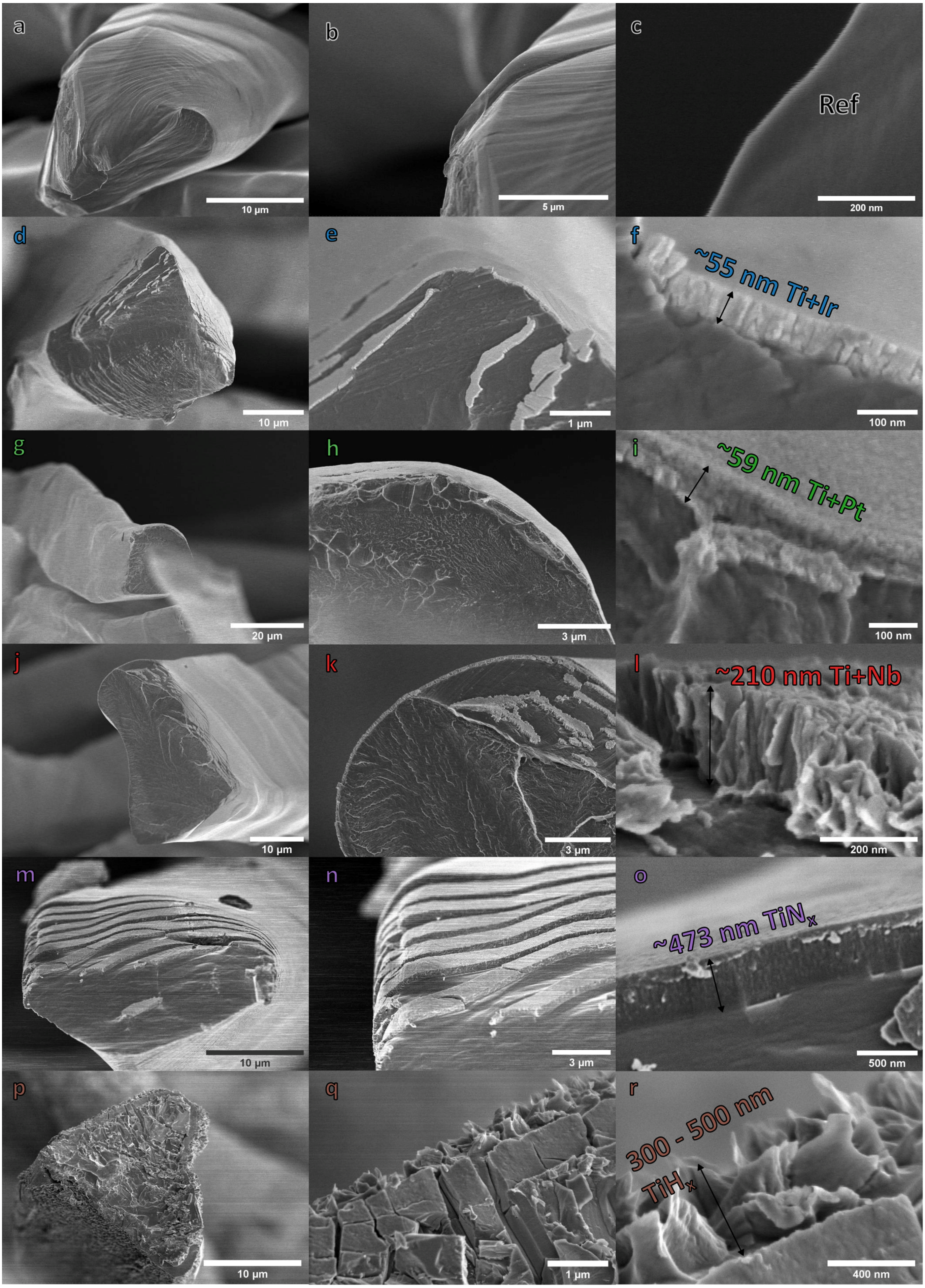

Figure S1. Different magnifications of cross-section SEM images for PTLs Ref (a-c), Ir (d-f), Pt (g-i), Nb (j-l), TiN_x (m-o) and TiH_x (p-r).

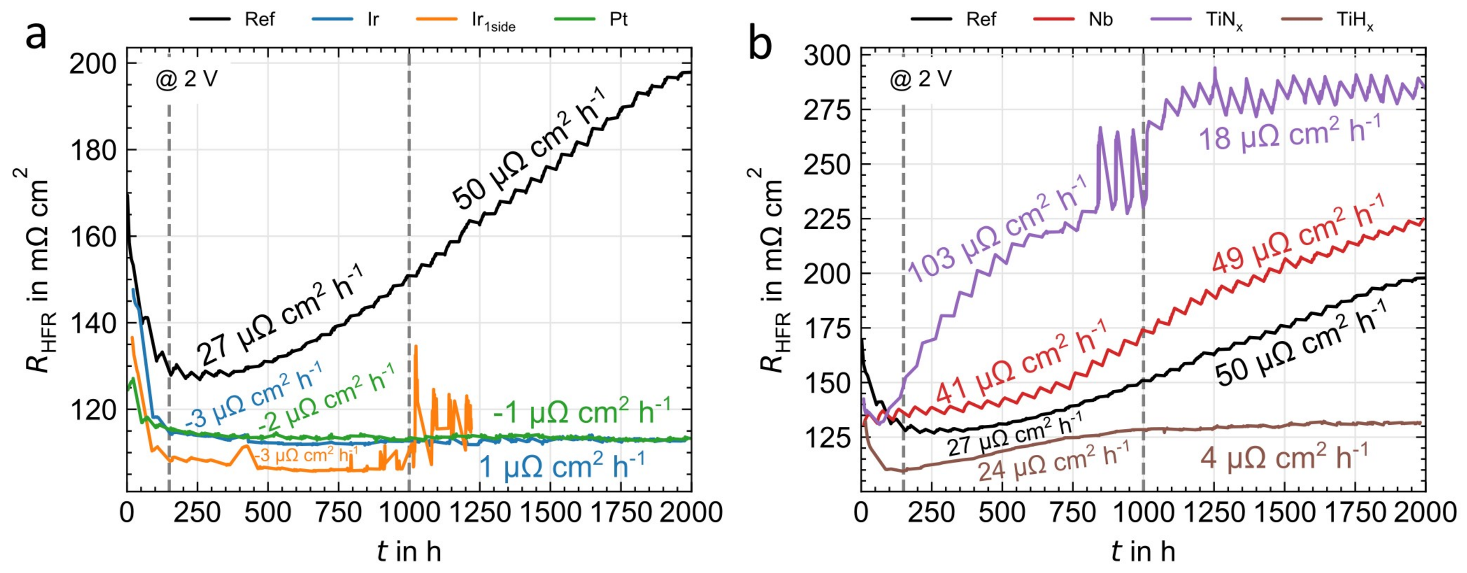

Figure S2. HFR over time for 2000 h long-term measurements with PGM (a) and PGM-free coatings (b) including degradation rates in μΩ cm^2 h^{-1}, extracted from 2 V steps of stress phases. For the TiN_x sample, mistakes in the HFR determination occur starting from about 800 h due to a shift of the Nyquist plot so a low frequency datapoint is estimated as the HFR because of a lower imaginary part in the impedance compared to the highest frequencies.

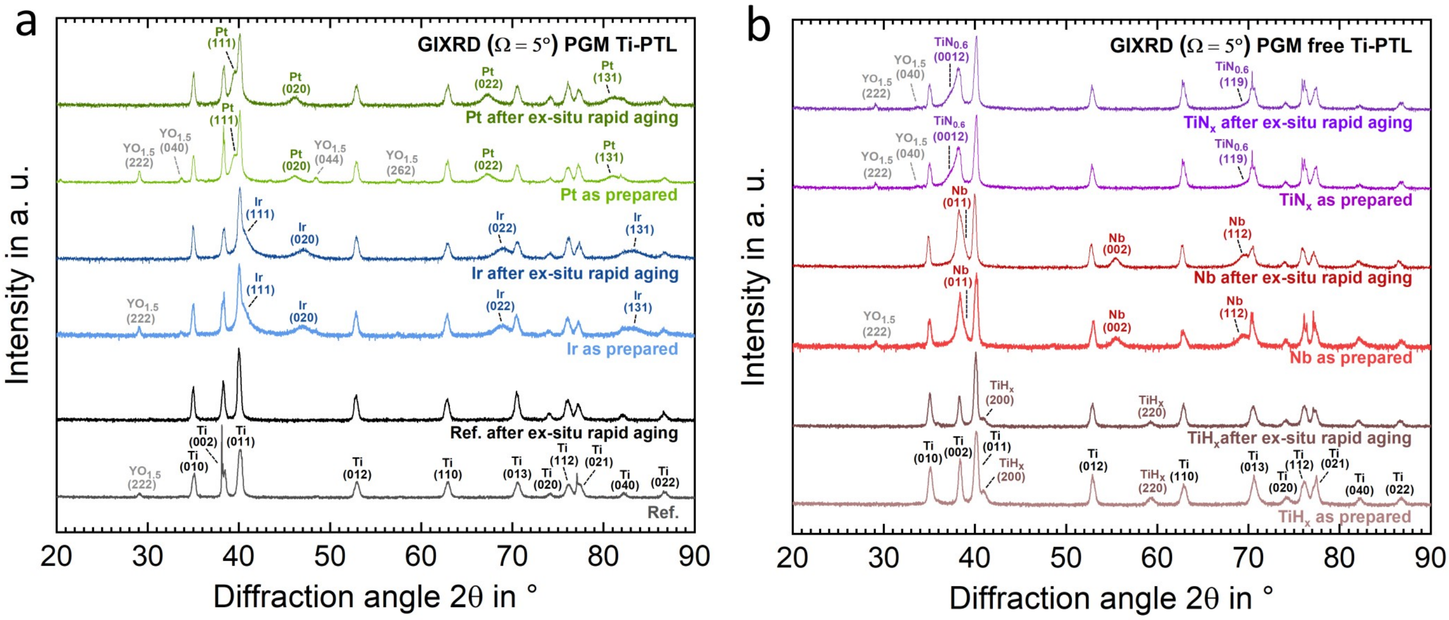

Figure S3. GIXRD measurements as prepared and after ex-situ rapid aging test for PGM coatings and Ref (a) as well as PGM-free coated PTLs (b).

The GIXRD spectra in (a) display no changes before and after the ex-situ rapid aging test in the typical X-ray reflections of the Ti[1], the corresponding Ir[1] (COD database code: 9008517) or Pt phase[2] (COD database code: 1011109). In the case of the PGM-free PTLs, no significant changes in the typical X-ray reflections of the TiH_x[3,4], Nb[5] (COD database code: 4000948) and $TiN_{0.6}$ phase[6] (COD database code: 1100040) can be seen (b). It should be noted that the TiH_x results contain an additional variation compared to all other samples due to the changed surface structure, which also exists in all pores and in areas shaded for coatings.

The X-ray reflections of the $YO_{1.5}$ phase[7] (COD database code: 1009013) can be seen in the GIXRD spectra before the ex-situ rapid aging test caused by particle contamination on the PTL surface. Only the hydrochloric acid treatment for producing the TiH_x successfully removed the YO_x particles before the test. After the test, the X-ray reflections of the $YO_{1.5}$ phase disappear from all PTLs except for the TiN_x coated PTL.

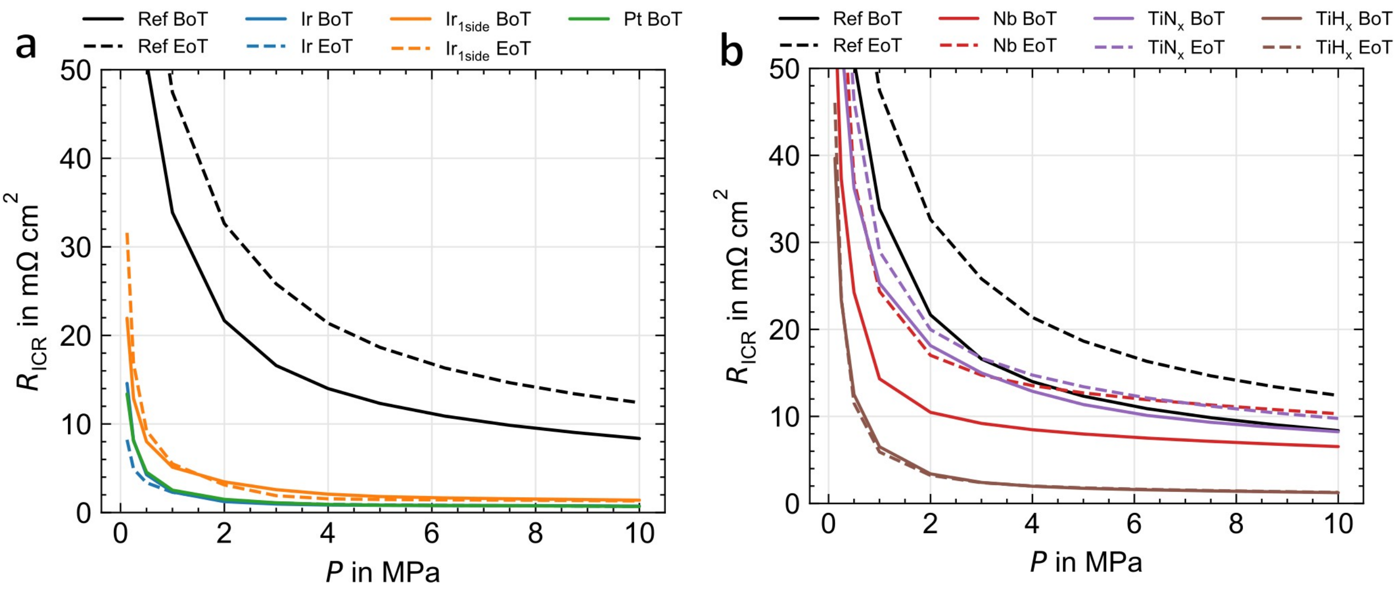

Figure S4. Universal testing machine contact resistance measurements over the whole pressure range for PGM (a) and PGM-free coatings (b) at begin (BoT) and end of the long-term test (EoT). A clamping pressure of 6.25 MPa was chosen for the bar plots in the manuscript. The procedure is described in the Experimental section of the manuscript.

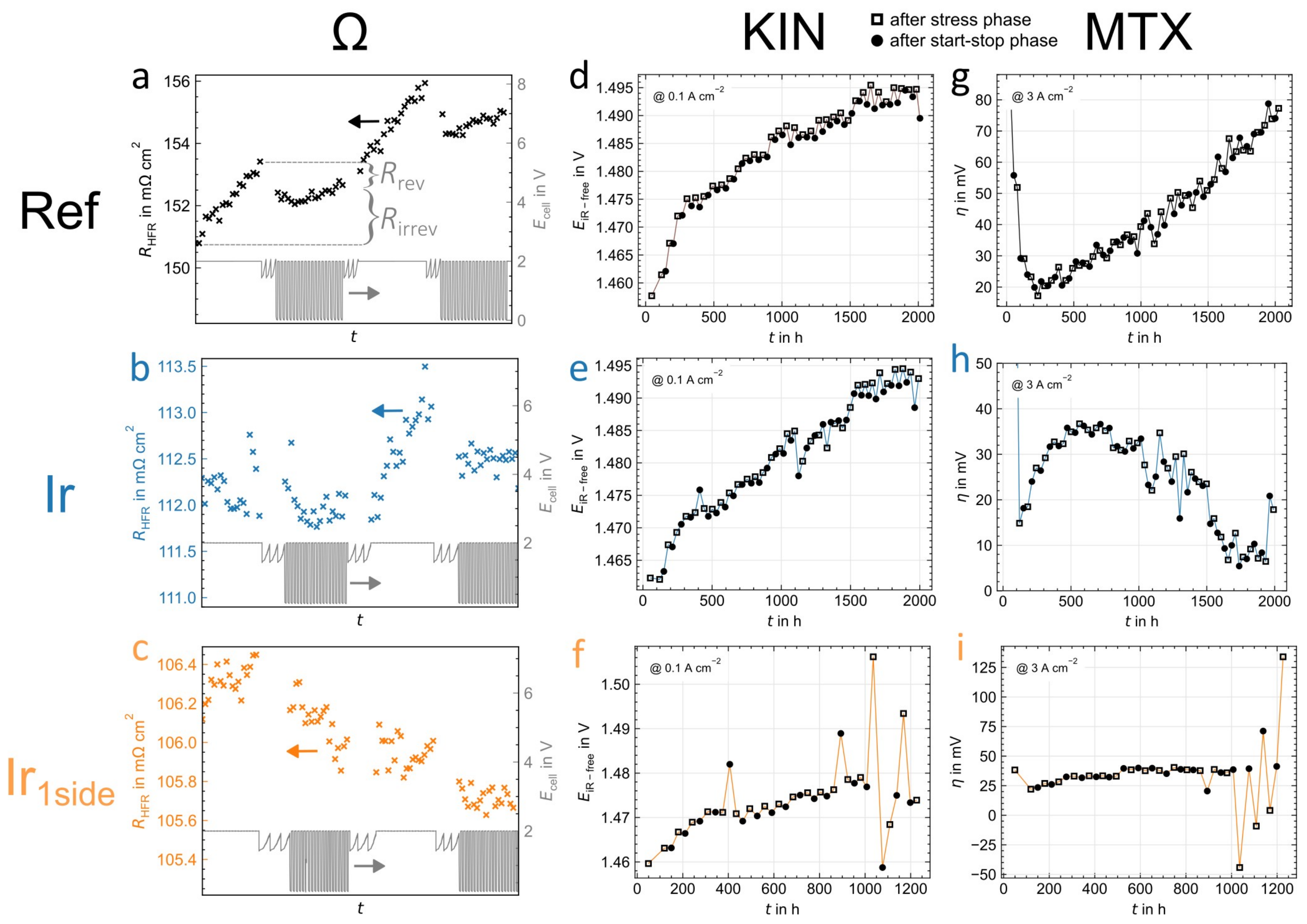

Figure S5. Reversible degradation of Ref and Ir coated PTLs. a-c: Ohmic reversible (R_{rev}) and irreversible (R_{irrev}) degradation in a roughly 120 h wide cut-out of HFR values over time. The corresponding protocol phases (stress phase, characterization, start-stop phase and second characterization) are visualized by the cell voltage in the lower part, while no HFR values are plotted for the characterization curves. d-f: kinetic degradation displayed as the HFR-free voltage at 0.1 A cm^{-2} extracted from polarization curves over the full testing time after stress (squares) and start-stop phases (circles). g-i: mass transport plus residual overvoltages at 3 A cm^{-2} extracted from polarization curves over the full testing time after stress (squares) and start-stop phases (circles).

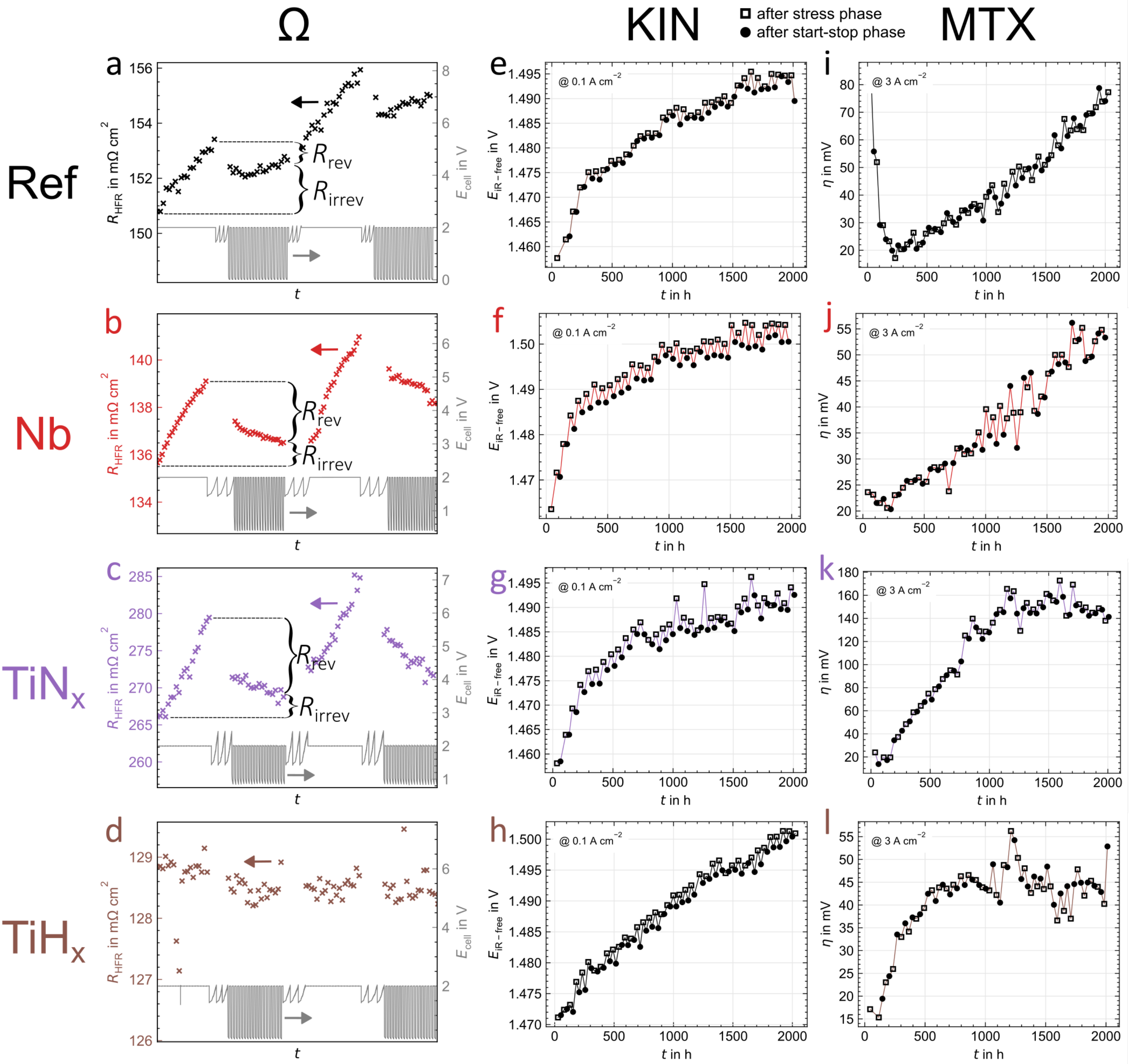

Figure S6. Reversible degradation of PGM-free coated PTLs. a-d: Ohmic reversible (R_{rev}) and irreversible (R_{irrev}) degradation in a roughly 120 h wide cut-out of HFR values over time. The corresponding protocol phases (stress phase, characterization, start-stop phase and second characterization) are visualized by the cell voltage in the lower part, while no HFR values are plotted for the characterization curves. e-h: kinetic degradation displayed as the HFR-free voltage at 0.1 A cm^{-2} extracted from polarization curves over the full testing time after stress (squares) and start-stop phases (circles). i-l: mass transport plus residual overvoltages at 3 A cm^{-2} extracted from polarization curves over the full testing time after stress (squares) and start-stop phases (circles).

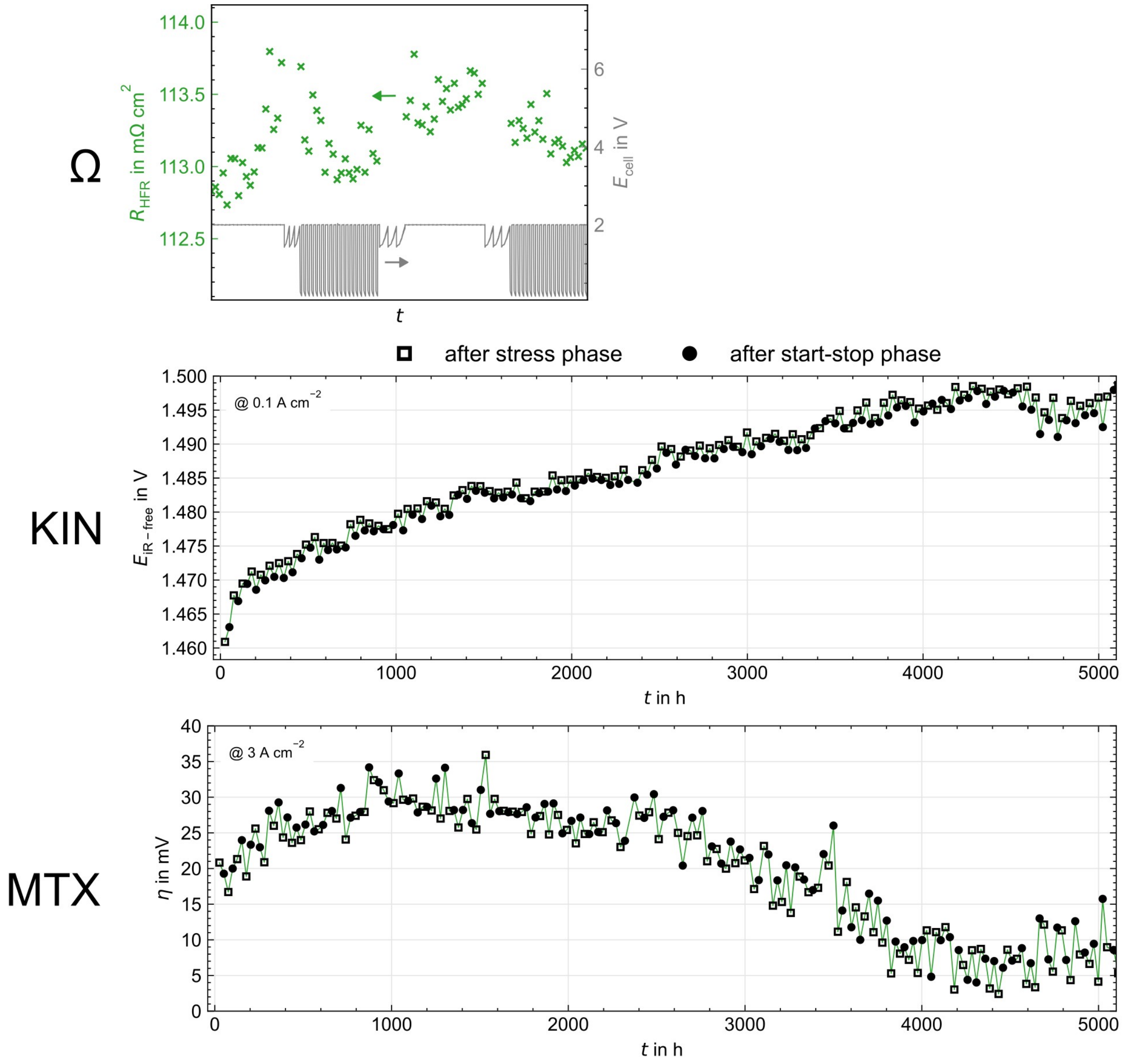

Figure S7. Reversible degradation of the Pt coated PTL. Ohmic degradation in a roughly 120 h wide cut-out of HFR values over time. The corresponding protocol phases (stress phase, characterization, start-stop phase and second characterization) are visualized by the cell voltage in the lower part, while no HFR values are plotted for the characterization curves. Kinetic degradation is displayed as the HFR-free voltage at 0.1 A cm^{-2} extracted from polarization curves over the full testing time after stress (squares) and start-stop phases (circles). Mass transport plus residual overvoltages at 3 A cm^{-2} are extracted from polarization curves over the full testing time after stress (squares) and start-stop phases (circles).

References

(1) Zemann, J. Crystal structures, 2 nd edition. Vol. 1 by R. W. G. Wyckoff. *Acta Cryst* **1965**, *18* (1), 139. DOI: 10.1107/S0365110X65000361.

(2) Hull, A. W. X-Ray Crystal Analysis of Thirteen Common Metals. *Phys. Rev.* **1921**, *17* (5), 571–588. DOI: 10.1103/PhysRev.17.571.

(3) Bautkinova, T.; Utsch, N.; Bystron, T.; Lhotka, M.; Kohoutkova, M.; Shviro, M.; Bouzek, K. Introducing titanium hydride on porous transport layer for more energy efficient water electrolysis with proton exchange membrane. *Journal of Power Sources* **2023**, *565*, 232913. DOI: 10.1016/j.jpowsour.2023.232913.

(4) Wu, T.-I.; Wu, J.-K. Effects of electrolytic hydrogenating parameters on structure and composition of surface hydrides of CP-Ti and Ti–6Al–4V alloy. *Materials Chemistry and Physics* **2002**, *74* (1), 5–12. DOI: 10.1016/S0254-0584(01)00403-5.

(5) Ling, C. D.; Avdeev, M.; Kutteh, R.; Kharton, V. V.; Yaremchenko, A. A.; Fialkova, S.; Sharma, N.; Macquart, R. B.; Hoelzel, M.; Gutmann, M. Structures, Phase Transitions, Hydration, and Ionic Conductivity of Ba 4 Nb 2 O 9. *Chem. Mater.* **2009**, *21* (16), 3853–3864. DOI: 10.1021/cm901644e.

(6) Lengauer, W.; Ettmayer, P. The crystal structure of a new phase in the titanium-nitrogen system. *Journal of the Less Common Metals* **1986**, *120* (1), 153–159. DOI: 10.1016/0022-5088(86)90637-5.

(7) Baldinozzi, G.; Bérar, J. F.; Calvarin-Amiri, G. Rietveld Refinement of Two-Phase Zr-Doped Y_2O_3. *MSF* **1998**, *278-281*, 680–685. DOI: 10.4028/www.scientific.net/MSF.278-281.680.
